# Limited-Data Radar–Audio Fusion for Room-Level Intrusion Detection: Multi-Level Performance Evaluation and Cross-Environment Validation

**DOI:** 10.3390/s26217095

**Published:** 2026-11-09

**Authors:** Eduard Vladu, Luminita Rostas, Marius Neag, Alessandro Battigelli, Dominic Domnici, Gabriel Oltean

**Affiliations:** Department of Bases of Electronics, Faculty of Electronics, Telecommunications and Information Technology, Technical University of Cluj-Napoca, Memorandumului Street No. 28, 400114 Cluj-Napoca, Romania; luminita.rostas@bel.utcluj.ro (L.R.); alessandro.battigelli@bel.utcluj.ro (A.B.); domnici.va.dominic@student.utcluj.ro (D.D.); gabriel.oltean@bel.utcluj.ro (G.O.)

**Keywords:** indoor intrusion detection, radar–audio fusion, multimodal sensing, camera-free monitoring, edge AI, stateful alarm evaluation, mmWave radar, acoustic sensing

## Abstract

**Highlights:**

**What are the main findings?**

• Radar and audio provide complementary information for room-level intrusion-related alarm detection, with score-level fusion offering a robust multimodal decision strategy.
• The frozen pipeline retained effective detection after transfer to a second room and sustained live sensor-to-decision operation on Raspberry Pi 5 within the 0.8 s update interval, although nuisance-alarm behavior remained environment-sensitive.

**What are the implications of the main findings?**

• The proposed self-contained configuration combines lightweight edge processing with a limited sensing hardware footprint, supporting cost-conscious deployment without dedicated sensors at individual doors and windows.
• The lightweight processing chain is compatible with edge deployment while explicitly addressing the trade-off between detection sensitivity and nuisance-alarm suppression.

**Abstract:**

Camera-free indoor security requires privacy-preserving sensing robust to disturbances and compatible with edge deployment. This study presents a limited-data radar–audio framework for room-level intrusion-related alarm detection. Data were collected using two colocated Infineon CY8CKIT-062S2-AI boards providing 60 GHz radar macro-detection outputs and stereo audio. Radar-only, audio-only, feature-level early-fusion, and score-level late-fusion branches were evaluated using session-disjoint training, validation, and test subsets, followed by stateful alarm processing. On the independent Room 1 test subset, validation-selected maximum-score late fusion achieved an F1-score of 0.897, compared with 0.842 for radar-only, 0.789 for audio-only, and 0.854 for early fusion. Stateful processing reduced false positives from 4.19 to 2.58 per 100 negative windows while retaining an F1-score of 0.888. Raspberry Pi 5 replay preserved all reference decisions. A live proof-of-concept sustained early-fusion and stateful maximum-score late-fusion processing at 48.53 ms and 46.50 ms per window, respectively, below the 0.8 s update interval. Without retraining or parameter adaptation, transfer to a second room retained F1-scores of 0.864 at window level and 0.859 after stateful processing, although false-positive behavior was more environment-sensitive. These results support score-level radar–audio fusion for limited-data edge-oriented indoor intrusion detection.

## 1. Introduction

Intrusion detection in residential, office, and restricted-access environments remains a technical challenge, particularly when monitoring must be continuous, camera-free, and compatible with low-cost edge deployment. A self-contained room-level sensing system is particularly attractive in this context because it can avoid the need for dedicated detection sensors at individual doors and windows. In small, protected rooms, an effective system must react promptly, tolerate environmental disturbances, and avoid the privacy burden of camera-based surveillance. These requirements motivate the use of non-contact multimodal sensing. Multisensor fusion provides a principled framework for combining heterogeneous evidence. In indoor monitoring, this complementarity can improve reliability and context awareness without relying on explicit visual identification [1].

Radar is well suited to room-level monitoring because it operates independently of visible illumination and does not capture conventional images. Radar-based human detection has consequently been investigated in security, surveillance, healthcare, and privacy-sensitive indoor monitoring [2,3,4]. The 60 GHz BGT60TR13C radar family is especially relevant to compact embedded systems because it supports contactless motion sensing in a small hardware footprint [3]. However, radar observations may remain ambiguous when human motion is very slow, occurs near the sensing boundary, or resembles non-intrusive motion, including motion of lightweight objects caused by heating or air-conditioning airflow.

Audio provides complementary information about footsteps, impacts, attempted access, object motion, and other context-dependent events. Previous studies have shown that acoustic sensing can support indoor intrusion detection under both line-of-sight and non-line-of-sight conditions [5], and that lightweight Internet of Things (IoT) implementations can distinguish intrusion-related sounds from events such as speech, keys, falling objects, and background noise [6]. At the same time, audio is affected by room acoustics, external sounds, environmental noise, and events that are acoustically salient but not security relevant. Radar and audio therefore exhibit different failure modes: an event may be weak in one modality while remaining detectable in the other.

Prior intrusion-monitoring systems further indicate that the problem should be treated as a sensing-and-decision pipeline rather than as an isolated classifier. Hybrid intrusion-monitoring systems have combined sound sensing with vision [7], while more recent embedded multimodal approaches have fused complementary sensing modalities, such as seismic and acoustic signals, for real-time intrusion detection [8]. Broader multimodal studies similarly show that heterogeneous sensors can compensate for one another when one channel becomes unreliable [9], and that fusion can reduce false alarms and improve decision robustness in surveillance applications [10]. Nevertheless, radar–audio fusion remains weakly represented for small-room indoor intrusion alarms, particularly when only a limited amount of application-specific training data is available. Limited data availability is also a recognized difficulty in indoor predictive modeling. Njima et al. [11] addressed this issue in indoor localization, investigating strategies based on generative data augmentation and semi-supervised learning to mitigate the limitations associated with restricted labeled training data. Although the target application differs from intrusion detection, their study highlights the broader challenge of developing reliable indoor models when only a limited amount of labeled data is available. In the present work, this constraint is addressed from a different perspective, by using compact modality-specific representations, lightweight classifiers, and session-disjoint development and evaluation subsets.

A second gap concerns evaluation. Many studies report performance primarily at the level of independent classifier outputs, although a deployed security system is judged by the alarms it produces over time. In resource-constrained acoustic monitoring, temporal aggregation and post-processing are required because local predictions may be fragmented or unstable [12]. Consequently, window-level discrimination and operational alarm behavior should be evaluated separately. Temporal smoothing, hysteresis, and persistence may reduce nuisance activations, but they can also delay or suppress true alarms; this trade-off cannot be inferred from window-level accuracy alone.

The present study addresses these gaps using an application-driven radar–audio dataset acquired in two protected indoor environments of comparable size. The main development dataset was collected in Room 1, an approximately 5 × 5 m room, using two colocated Infineon CY8CKIT-062S2-AI boards, while an additional set of recordings was subsequently acquired in Room 2 for external cross-environment evaluation. The acquisition used 60 GHz radar macro-detection outputs at 10 Hz and stereo audio at 16 kHz.

The target is formulated as an intrusion-related alarm event rather than as a generic activity class or a strict occupancy label. Positive examples include unauthorized entry and alarm-relevant access interactions associated with an intrusion process, while negative examples include external motion, ambient acoustic clutter, siren-like sounds without intrusion, speech without intrusion, and falling objects of different types. The task is therefore binary but not trivial: its difficulty arises from hard negatives, heterogeneous sensor responses, and the possibility that a genuine alarm event is expressed strongly in only one modality.

Four detection branches are compared under the same event definition and the same session-disjoint data partition: radar-only, audio-only, feature-level early fusion, and score-level late fusion. Compact modality-specific representations and lightweight classifiers are used to maintain low model complexity under limited-data conditions. Performance is evaluated first at the window level and subsequently through a stateful alarm mechanism based on temporal smoothing, separate activation and deactivation thresholds, and persistence constraints. This two-stage evaluation explicitly distinguishes local classification performance from the stability and sensitivity of the resulting sequential alarm output.

The primary methodological contribution of this work is the integration of compact radar–audio representations, fusion-stage comparison, and stateful alarm generation within a common limited-data, edge-oriented evaluation framework. Rather than considering multimodal fusion only as an independent classification problem, the proposed methodology follows the complete progression from synchronized sensor observations to window-level multimodal scores and finally to operational alarm decisions. This distinguishes the present approach from related indoor monitoring designs based on single modalities, other sensor combinations, or evaluations limited primarily to independent classifier outputs. More specifically, the study: (1) evaluates the complementarity of radar and audio using a modality-independent alarm definition and strict session-level separation; (2) directly compares unimodal, feature-level early-fusion, and score-level late-fusion strategies using the same aligned observations; (3) quantifies how temporal alarm processing changes the balance between detection sensitivity and nuisance-alarm suppression; (4) evaluates edge deployment through both replay-based benchmarking and live multimodal sensor-to-decision execution on a Raspberry Pi 5; and (5) evaluates the complete frozen processing pipeline after relocation to a second room of comparable size but different internal geometry, construction, and furnishing materials, without retraining or environment-specific parameter adaptation.

## 2. Materials and Methods

### 2.1. Hardware and Data Acquisition Setup

This subsection describes the sensing hardware, room-level acquisition setup, board placement, and practical acquisition configuration used to build the radar–audio intrusion dataset. The acquisition system was built around the CY8CKIT-062S2-AI Edge AI evaluation platform (Infineon Technologies AG, Neubiberg, Germany), which integrates radar and audio sensing and is therefore well suited to camera-free multimodal indoor monitoring under embedded-system constraints [13].

The selected sensing hardware also supports a cost-conscious implementation. The radar and acoustic devices used in the experimental platform are commercially available as standalone components and could be integrated on a dedicated sensing PCB. Such an implementation would avoid the cost and redundancy associated with using development boards in a final system.

The sensing setup combines a 60 GHz onboard radar channel and an onboard stereo audio channel. In the configuration adopted for this work, the radar stream was operated in macro-detection mode with an effective output update rate of 10 Hz, while audio was recorded in stereo at 16 kHz. DeepCraft Studio, version 5.8.4292.0 (Imagimob AB, Stockholm, Sweden) was used to support event annotation and to define the temporal intervals associated with the recorded scenarios. Subsequent preprocessing, radar–audio temporal alignment, feature preparation, and dataset organization for model development and evaluation were performed in Google Colab (Google LLC, Mountain View, CA, USA). This workflow allowed the two sensing modalities to be processed independently while preserving a common event-level ground truth for multimodal evaluation.

Radar was included because it provides contactless motion sensing without relying on visible-light imaging, which is advantageous in privacy-sensitive indoor monitoring scenarios. The analysis therefore focuses on the macro-detection signal exported at 10 Hz rather than on a full low-level frequency-modulated continuous-wave (FMCW) processing chain. This compact representation is consistent with the edge-oriented character of the proposed system. Detailed characteristics of the onboard radar device are provided in [14].

Audio was acquired concurrently to provide complementary information about intrusion-related events at low sensing complexity. In real-life indoor environments, acoustic observations may capture footsteps, impacts, object motion, external alarm sounds, and other context-dependent cues that are not necessarily expressed with the same salience in radar data. Here, stereo audio sampled at 16 kHz provided sufficient temporal resolution for the considered intrusion-related events while remaining compatible with lightweight downstream processing.

Experiments were performed in Room 1, a restricted-access room of approximately 5 × 5 m, with a single door located on the wall opposite the acquisition viewpoint and a window on the left-side wall. Since the acquisition workflow used in this study required the radar stream to be handled separately from the audio stream, two closely colocated CY8CKIT-062S2-AI boards were operated simultaneously during recording. Figure 1 illustrates the Room 1 layout and the main monitored region.

The sensing position was located approximately at the center of the wall opposite the door, such that the sensing direction covered the principal door-side region while also maintaining lateral visibility toward the windowed side of the room. This placement provided coverage of the main access path while preserving sensitivity to lateral activity relevant to intrusion decisions. The two evaluation boards were closely colocated with a small spatial offset, allowing both sensing units to observe approximately the same scene.

### 2.2. Dataset Construction and Event Definition

The radar–audio dataset used in this study was custom-collected for edge-oriented intrusion-related alarm detection and is publicly available in [15]. The complete public release contains 80 recording sessions acquired in two indoor environments. Seventy Room 1 sessions were used for model development and primary evaluation, whereas ten additional Room 2 sessions were reserved exclusively for external cross-environment evaluation. The release contains the radar and audio recordings, session-level organization, and event annotations used throughout the study. Although the final formulation is binary, the acquisition protocol was intentionally designed to expose the sensing system to both diverse intrusion-related events and realistic non-intrusive disturbances that may trigger false alarms in practical deployments. The resulting dataset is therefore not intended as a large-scale benchmark, but as an application-driven multimodal testbed tailored to camera-free indoor security under constrained sensing and deployment conditions.

The positive class was defined operationally as an intrusion-related alarm event. The core target corresponds to unauthorized access to the secured room, but the positive label set also includes alarm-relevant boundary interaction patterns when they occur as part of the intrusion process, such as strong door impacts and attempted opening actions involving the handle, key, or lock region. These events were not annotated as a separate precursor or attempted-entry class. Accordingly, the positive label should be interpreted as an alarm-oriented security label, rather than as a strictly physical room-occupancy label.

Positive events include single-intruder entry, two-intruder entry, and rear-side intrusion initiation, corresponding to cases where the subject appears from the rear sensor side rather than through the main front-facing access direction. Motion execution within positive sequences was intentionally varied to include fast motion, slow motion, and stealth-like motion, in order to broaden the temporal and kinematic variability observed by the sensing system. In addition, positive events were recorded both in quiet conditions and in the presence of ambient perturbations, and some sequences also include speech produced by the intruder(s). This design reduces the risk that the models learn an overly narrow association between intrusion and a specific motion pace or acoustic context. The negative class was designed to include several hard negatives relevant to room-level security. These include falling objects of different sizes and materials, such as boxes, books, cups, and metallic, plastic, or wooden objects; air-conditioner noise; urban/environmental noise, including outdoor nuisance sounds such as construction-related activity and vehicle horns; storm and rain noise; external siren-like acoustic events; and human voices without intrusion. Negative events therefore include both indoor non-intrusive disturbances and external acoustic confounders. Together, these cases make the task substantially more realistic than a clean silent-room intrusion-versus-no-intrusion setup.

Data were acquired in recording sessions and subsequently converted into synchronized radar–audio samples through the windowing procedure described in Section 2.3. To avoid leakage between training and evaluation, the dataset was partitioned at the session level, rather than by randomly splitting individual windows. Thus, windows originating from the same recording were kept within the same subset. This group-aware partitioning is particularly important in the present study because neighboring windows from the same session are strongly correlated in both motion and acoustic structure.

The 70 Room 1 recording sessions were assigned to training, validation, and test subsets at the session level, using 42, 14, and 14 sessions, respectively. A fixed random seed was used to initialize the session assignment, which was subsequently refined at the session level to obtain comparable positive-window proportions across the three subsets while ensuring sufficient positive representation in the validation and test subsets. No individual windows were transferred independently between subsets. Once the session assignment was established for the radar recordings, the same assignment was applied unchanged to the audio modality. Window generation and radar–audio temporal alignment were then performed within the resulting fixed session subsets. The final session-disjoint partition comprised 42 training sessions, 14 validation sessions, and 14 test sessions, with the aligned class distribution reported in Table 1.

The aligned dataset was used for early fusion, late fusion, and the common comparison of the four detection branches. In particular, all reported test results were computed on the same set of 1244 aligned windows, containing 624 positive and 620 negative samples. This common evaluation subset ensures that differences between radar-only, audio-only, early-fusion, and late-fusion performance are not caused by differences in test-set composition. Paired windows had a median absolute temporal-center difference of approximately 50 ms. Two training pairs and two validation pairs initially showed modality-specific label disagreement near event boundaries. In these cases, the common event-level ground-truth annotation was retained for both modalities. No label disagreement occurred in the test subset.

### 2.3. Multimodal Windowing and Preprocessing

The radar and audio recordings were organized by acquisition session and transformed into temporally corresponding samples before model training. The radar stream, available at 10 Hz, was segmented into windows of 16 consecutive frames with a stride of 8 frames. Each radar window therefore represents 1.6 s of observation, while successive windows are advanced by 0.8 s. A center-based assignment rule was used so that each window inherited the event label associated with its temporal center. This procedure limits rapid label oscillation close to transition boundaries while preserving sufficient temporal overlap for alarm tracking.

The audio stream was recorded in stereo at 16 kHz and converted to mono before feature extraction. Audio samples corresponding to the radar observation interval were selected from the same recording session. The acoustic representation was based on a log-mel analysis using 64 mel bands, a 1024-point fast Fourier transform, an analysis window of 1024 samples, and a hop length of 256 samples. This representation retains short-duration impact and footstep information while providing a compact time–frequency description suitable for lightweight learning models.

Radar and audio preprocessing were performed independently before fusion. Modality-specific transformations, clipping limits, and scaling statistics were estimated exclusively from the training data and then reused unchanged for validation and test data. This separation prevents information leakage and avoids allowing the larger numerical range of one modality to dominate the fused representation. For fixed-length classifiers, the processed radar and audio windows were summarized into compact modality-specific feature vectors. The aligned vectors were stored together with the recording-session identifier, temporal order, and common intrusion-related alarm label.

Temporal correspondence between modalities was maintained within each recording session. Only radar and audio windows representing the same acquisition interval were paired for early fusion. This group-aware alignment is important because the two streams have different native sampling rates and because random pairing could create artificially easy or physically inconsistent samples. The same alignment map was retained for training, validation, test, and replay-based deployment evaluation.

### 2.4. Unimodal and Multimodal Detection Framework

Four detection paths were evaluated using the same temporally aligned radar–audio windows, the same intrusion-related alarm definition, and the same session-disjoint data partition, as summarized in Figure 2. The radar-only and audio-only branches, shown in Figure 2a,b, process the two sensing modalities independently and provide the unimodal reference results. The early-fusion branch in Figure 2c combines temporally aligned compact radar and audio representations at the feature level before classification, whereas the late-fusion branch in Figure 2d combines the window-level scores produced by the independently trained unimodal classifiers. This organization enables a direct comparison between unimodal processing, feature-level fusion, and score-level fusion under the same limited-data experimental conditions. In Figure 2, short-time Fourier transform (STFT) is used in the audio preprocessing branch, while average-score fusion (AVG), maximum-score fusion (MAX), product-score fusion (PROD), and logistic-regression fusion (LR) denote the four late-fusion strategies. NFFT denotes the number of fast Fourier transform points used in the STFT computation.

**Figure 2:**
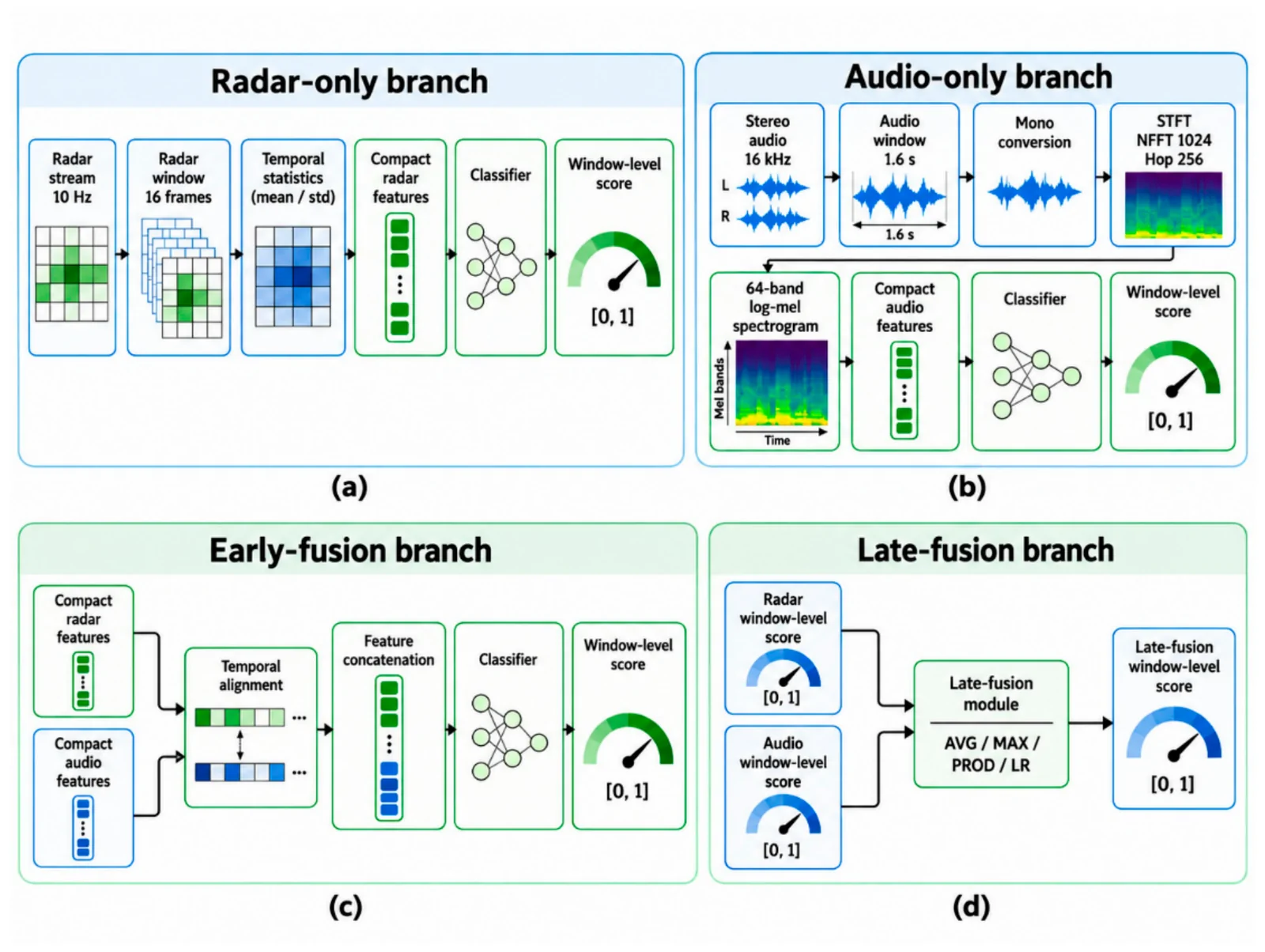
Processing pipelines for: (**a**) radar-only detection; (**b**) audio-only detection; (**c**) feature-level early fusion; and (**d**) score-level late fusion.

#### 2.4.1. Radar-Only Detection Branch

The radar-only processing path is illustrated in Figure 2a. Each radar sample contains 16 consecutive macro-detection frames acquired at 10 Hz and therefore represents a 1.6 s observation interval. Since each frame contains 2048 values, direct vectorization would produce 32,768 input values per window, which is unnecessarily large relative to the limited number of independent recording sessions.

Rather than adopting a higher-capacity learned representation, the radar window was summarized using simple deterministic temporal statistics to limit dimensionality and reduce the risk of overfitting given the limited number of independent recording sessions. Temporal mean and standard deviation were selected to retain complementary information about the average radar response and its temporal variability.

Each radar window was therefore summarized through temporal statistics. For the i-th window, the temporal mean was calculated independently at each radar sample index *j*:

(1) $$\mu_{r,i}\left(j\right)=\;\frac{1}{T_{r}}\sum_{t=1}^{T_{r}}R_{i}\left(t,j\right),\;j=1,\ldots ,N_{r}$$

where $T_{r}=16$ is the number of frames in the window, $N_{r}=2048\;$ is the number of radar values per frame, and $R_{i}\left(t,j\right)\;$ denotes the value at frame *t* and radar sample index j. The corresponding temporal standard-deviation profile was obtained as:

(2) $$\sigma_{r,i}\left(j\right)=\;\sqrt{\frac{1}{T_{r}}\sum_{t=1}^{T_{r}}{{(R}_{i}\left(t,j\right)-\mu_{r,i}\left(j\right))}^{2}}$$

The mean profile captures the average radar response within the observation interval, whereas the standard-deviation profile represents temporal variation associated with motion and scene changes. To further reduce dimensionality, both profiles were average-pooled over non-overlapping groups of adjacent values:

(3) $${\bar{\mu }}_{i,k}^{\left(r\right)}=\;\frac{1}{B}\sum_{j=\left(k-1\right)B+1}^{kB}{µ}_{i,j}^{\left(r\right)},\;{\bar{\sigma }}_{i,k}^{\left(r\right)}=\;\frac{1}{B}\sum_{j=\left(k-1\right)B+1}^{kB}\sigma_{i,j}^{\left(r\right)},\;\;$$

where *B* = 8 is the pooling factor and *k* = 1, …, *N*_r_/*B* = 256 indexes the pooled radar sample groups. The pooling is applied to adjacent sample indices in the flattened radar acquisition representation and therefore does not correspond to a fixed physical distance in the monitored room. The pooled mean and standard-deviation values form two 256-dimensional profiles, ${\bar{\mu }}_{i,k}^{\left(r\right)}\;and$ ${\bar{\sigma }}_{i,k}^{\left(r\right)}$ respectively. These profiles were concatenated to obtain the compact radar representation $x_{i}^{\left(r\right)}=[{\bar{\mu }}_{i,k}^{\left(r\right)}\;;$ ${\bar{\sigma }}_{i,k}^{\left(r\right)}]\in R^{512}$. Thus, each radar window was reduced from 32,768 raw values to 512 features while retaining information on both the average response and its temporal variability. The resulting vector was then provided to the radar-only classifier, which produced the window-level intrusion score $s_{i}^{\left(r\right)}$. Because pooling is applied after computing the temporal mean and standard-deviation profiles, temporal motion variability is retained, while local variations across adjacent radar samples are smoothed.

#### 2.4.2. Audio-Only Detection Branch

The audio-only processing path is illustrated in Figure 2b. The original stereo recordings, acquired at 16 kHz, were divided into 1.6 s observation windows corresponding to the radar window duration and converted to mono before acoustic feature extraction. Each audio window was transformed into a time–frequency representation using the STFT, with an FFT length and analysis window of 1024 samples and a hop length of 256 samples. The spectral representation was projected onto 64 mel-frequency bands and logarithmically compressed to obtain the log-mel representation:

(4) $$M_{i}\;(m,\tau )=log\left(\sum_{k}H_{m}\;\left(k\right){|X_{i}\;(k,\tau )|}^{2}+\varepsilon \right)$$

where $X_{i}\;\left(k,\tau \right)\;$ denotes the STFT coefficient of audio window i at frequency bin k and time frame τ, $H_{m}\;\left(k\right)$ denotes the m-th mel filter, m = 1, …, 64, and ε is a small positive constant used to avoid numerical instability in the logarithm.

(5) $$\mu_{i,m}^{\left(a\right)}=\frac{1}{T_{a}}\sum_{\tau =1}^{T_{a}}M_{i}\left(m,\tau \right),\;\sigma_{i,m}^{\left(a\right)}=\sqrt{\frac{1}{T_{a}}\sum_{\tau =1}^{T_{a}}{\left[M_{i}\left(m,\tau \right)-\mu_{i,m}^{\left(a\right)}\right]}^{2}}$$

where $T_{a}\;$ denotes the number of time frames in the log-mel representation. The temporal mean and standard-deviation profiles were concatenated to form the compact audio feature vector $x_{i}^{\left(a\right)}\;=[\mu_{i}^{\left(a\right)}\;;\sigma_{i}^{\left(a\right)}\;]$. Since 64 mel-frequency bands were used, the concatenated mean and standard-deviation profiles produced a 128-dimensional audio feature vector.

This statistical summarization reduces the dimensionality of the two-dimensional log-mel representation while retaining information on both the average spectral distribution and its temporal variability. The resulting compact vector was provided to the audio-only classifier, which generated the corresponding window-level intrusion score $s_{i}^{\left(a\right)}\;∊\;\left[0,1\right]$.

#### 2.4.3. Early-Fusion Detection Branch

The final early-fusion branch used temporally aligned compact radar and audio descriptors concatenated before classification. A higher-capacity dual-branch alternative was also evaluated during development but was not retained because its additional trainable complexity did not provide a stable performance advantage under the limited-data conditions of this study.

More specifically, the radar-only and audio-only preprocessing stages first produced a compact radar feature vector and a compact audio feature vector for each synchronized observation window. Let $x_{i}^{\left(r\right)}\;$ denote the radar feature vector associated with window i and let $x_{i}^{\left(a\right)}$ denote the corresponding audio feature vector extracted from the same time interval. Because both modalities were segmented using the same synchronized window definition, the two feature vectors were directly aligned in time and fused by concatenation:

(6) $$x_{i}^{\left(e\right)}=\left[\begin{array}{c} x_{i}^{\left(r\right)}\\ x_{i}^{\left(a\right)} \end{array}\right]$$

where *d_r_* = 512 and *d_a_* = 128 denote the radar and audio feature dimensions, respectively. Therefore, $x_{i}^{\left(e\right)}∊R^{640}\;$ represents the concatenated early-fusion feature vector associated with window i. The concatenated feature vector was then used as the input to the early-fusion classifier, which produced a window-level intrusion-related score:

(7) $$s_{i}^{\left(e\right)}=f_{e}(x_{i}^{\left(e\right)})$$

where $f_{e}()$ denotes the trained early-fusion decision model and $s_{i}^{\left(e\right)}\;∊\;\left[0,1\right]$ is the corresponding window-level score.

No point-wise spatial registration between the radar and audio representations was performed. The alignment used for early fusion was temporal, at the observation-window level, such that the radar and audio descriptors associated with a given sample originated from the same 1.6 s acquisition interval. The stereo audio was converted to mono before feature extraction; therefore, inter-channel spatial information was not retained in the 128-dimensional audio descriptor. Similarly, the 2048 radar values were not individually mapped to audio features. Instead, the radar stream was independently summarized into a 512-dimensional descriptor, which was subsequently concatenated with the 128-dimensional audio descriptor to form the 640-dimensional early-fusion vector.

Compared with the unimodal branches, this feature-level fusion strategy enables a joint decision from temporally aligned radar and audio descriptors. However, because each modality is summarized over the complete observation window using compact statistical features, fine within-window temporal correspondences between radar-observed motion and acoustic events are not preserved. This represents an intentional trade-off between temporal detail and model complexity under the limited-data, edge-oriented conditions considered here, and may partly explain why feature-level early fusion did not outperform score-level late fusion.

#### 2.4.4. Late-Fusion Detection Branch

The late-fusion processing path is illustrated in Figure 2d. In contrast to early fusion, radar and audio were processed independently up to the decision stage. The two unimodal classifiers therefore produced separate window-level intrusion scores, denoted by (s_i_^(r)^) for radar and (s_i_^(a)^) for audio. Fusion was subsequently performed directly on these scores, preserving the modality-specific decision functions while introducing only a lightweight combination stage.

Four score-level fusion strategies were evaluated. The first three were deterministic rules. The AVG rule assigns equal weight to the radar and audio scores:

(8) $$s_{i}^{\left(AVG\right)}=\frac{\left[s_{i}^{\left(r\right)}+s_{i}^{\left(a\right)}\right]}{2}$$

The MAX rule retains the strongest intrusion evidence produced by either modality:

(9) $$s_{i}^{\left(MAX\right)}=\max\left(s_{i}^{\left(r\right)},\;s_{i}^{\left(a\right)}\right)$$

The PROD rule combines the two scores multiplicatively:

(10) $$s_{i}^{\left(PROD\right)}=s_{i}^{\left(r\right)}\cdot s_{i}^{\left(a\right)}$$

The three deterministic rules reflect different assumptions about multimodal evidence. AVG provides a balanced contribution from both modalities, MAX favors detection whenever either radar or audio produces strong intrusion evidence, whereas PROD emphasizes agreement between the two unimodal classifiers by producing a high fused score primarily when both modality scores are high.

In addition to these fixed rules, an LR meta-model was evaluated to learn the relative contribution of radar and audio scores from the development data. The corresponding fused score was defined as:

(11) $$s_{i}^{\left(LR\right)}=\frac{1}{1+e^{-(\beta_{0}+\beta_{r}\cdot s_{i}^{\left(r\right)}+\;\beta_{a}\cdot s_{i}^{\left(a\right)})}}$$

where β_0_ is the intercept and β_r_ and β_a_ are the learned coefficients associated with the radar and audio scores, respectively.

All late-fusion strategies operate only on the two unimodal window-level scores and therefore require no additional high-dimensional multimodal feature representation. This makes the late-fusion stage computationally lightweight while allowing radar and audio evidence to remain independently modeled until the final decision stage. Such separation is particularly relevant in the present application because the two sensing modalities may provide complementary evidence and exhibit different failure modes under intrusion-related and nuisance conditions.

The late-fusion strategy and its corresponding decision threshold were selected using the validation subset and subsequently kept fixed for evaluation on the independent test subset. The resulting late-fusion window-level score was then used as the output of the late-fusion branch and, in the subsequent stateful evaluation, as the input to the temporal alarm-processing stage.

### 2.5. Model Training and Window-Level Evaluation

Multiple candidate classifiers were evaluated for the radar-only, audio-only, and early-fusion branches instead of selecting a single learning model a priori. The candidate set included a multilayer perceptron (MLP), logistic regression, histogram-based gradient boosting, Random Forest, Extra Trees, and a calibrated linear support vector classifier (Linear SVC). This comparison was intended to determine which classifier family provided the most suitable discrimination performance for each sensing representation under the limited-data conditions of the study. For late fusion, model development instead consisted of comparing the score-combination strategies described in Section 2.4.4.

Model development was performed using only the training and validation subsets. When cross-validation was used for model and hyperparameter selection, a stratified five-fold group-aware procedure was applied, with all windows originating from the same recording session kept within the same fold. This restriction prevents strongly correlated overlapping windows from the same physical recording from appearing simultaneously in the training and validation folds.

All preprocessing operations that required parameter estimation, including feature scaling and modality-specific normalization, were fitted exclusively using the corresponding training data and subsequently applied unchanged to validation data. The same principle was followed during cross-validation, such that preprocessing parameters were re-estimated independently within each training fold. Candidate classifier configurations and their hyperparameters were compared using development data only.

Model selection was performed separately for the radar-only, audio-only, early-fusion, and late-fusion branches. The primary selection criterion was the validation F1-score, while precision, recall, and false-positive behavior were also considered because missed intrusion-related events and nuisance alarms have different operational consequences. Consequently, the selected classifier family was not required to be identical across sensing branches.

After the classifier configuration had been fixed, the continuous decision threshold of each branch was selected from validation scores rather than being automatically set to 0.5. Threshold values were searched over the valid score range, with the objective of maximizing the validation F1-score while retaining the associated precision, recall, and false-positive characteristics. Neither model configuration nor decision threshold was subsequently modified using the independent test subset.

The test subset was therefore used only for the final window-level evaluation of the selected configurations. Performance was characterized using precision, recall, F1-score, receiver-operating-characteristic area under the curve (ROC-AUC), precision–recall area under the curve (PR-AUC), false-positive behavior, and confusion matrices. Candidate-model comparisons used for model selection are reported separately in the Section 3, whereas graphical analyses are restricted to the configurations retained after validation-based selection. False-positive behavior is additionally reported as FP/100, defined as the number of false-positive windows per 100 ground-truth negative windows.

### 2.6. Stateful Alarm Processing and Evaluation Methodology

Window-level scores were additionally processed using a stateful alarm mechanism intended to represent the temporal behavior of a practical intrusion-detection system. Unlike the window-level evaluation, in which each observation window is treated independently, the stateful evaluation preserves information from preceding windows and generates an alarm sequence through temporal smoothing, hysteresis, and persistence constraints. Let $p_{i}$ denote the intrusion-related score produced by the selected detection branch for window i. Temporal fluctuations were reduced using an exponential moving average (EMA):

(12) $$\tilde{p_{i}}=\alpha \cdot p_{i}+(1-\alpha )\cdot \tilde{p_{i-1}}$$

where $\tilde{p_{i}}$ is the smoothed score and α∊(0,1] controls the trade-off between rapid response and temporal stability. Larger values of α make the alarm more responsive to the current window, whereas smaller values provide stronger smoothing across consecutive observations. The smoothed score was evaluated using separate activation and deactivation thresholds, ${Ɵ}_{ON}$ and ${Ɵ}_{OFF}$, satisfying ${Ɵ}_{ON}\geq \;{Ɵ}_{OFF}$. When ${Ɵ}_{ON}$ > ${Ɵ}_{OFF}$, the resulting hysteresis band reduces rapid switching between alarm and non-alarm states when the score fluctuates near the decision boundary.

Persistence constraints were applied in addition to hysteresis. Alarm activation required the activation condition to remain satisfied for a predefined number of consecutive windows, denoted by $N_{ON}$, while deactivation required the release condition to persist for $N_{OFF}$ consecutive windows. Short isolated score excursions were therefore prevented from immediately changing the alarm state. Temporal alarm parameters were optimized exclusively on the Room 1 validation sequences using branch-specific exhaustive grid searches. The candidate values for the EMA coefficient, activation and deactivation persistence, and hysteresis band are summarized in Table 2. For each EMA coefficient, activation thresholds were generated from the unique empirical quantiles of the corresponding smoothed validation scores. The search used 401 quantile points for radar-only and audio-only detection and 301 for early fusion. For late fusion, two complementary optimization strategies were evaluated: a false-positive-prioritized search using 401 quantile points and an F1-prioritized search using 121 quantile points. In each case, the deactivation threshold was obtained by subtracting the selected hysteresis band from the activation threshold, with a lower bound of zero. All parameter searches and candidate selections were performed using validation data only.

The false-positive burst rate denotes the number of continuous false-alarm activations per 100 ground-truth negative windows, with bursts counted independently within each recording session.

Candidate configurations were first evaluated against the branch-specific feasibility constraints defined in Table 2. For radar-only and early-fusion detection, feasible candidates were ranked by lower FP/100, followed by higher recall, fewer false-positive bursts, higher precision, and higher F1-score. For audio-only detection, the false-positive burst rate was prioritized before recall. When no configuration satisfied both feasibility constraints, the smallest recall shortfall was prioritized, followed by lower FP/100 and fewer false-positive bursts.

For late fusion, two complementary validation strategies were considered. The false-positive-prioritized search favored configurations with lower FP/100 while maintaining the required recall, whereas the F1-prioritized search favored higher validation F1-score under the same feasibility constraints. The resulting feasible late-fusion candidates were then compared using a composite validation score combining F1-score, recall, precision, FP/100, and false-positive burst rate:

(13) $$S=0.40\cdot F1+0.25\cdot Recall+0.20\cdot Precision-0.10\cdot \frac{FP/100}{5}-0.05\cdot \frac{FPbursts/100}{5}$$

Model configuration and temporal alarm parameters were selected for each detection branch using validation data and were subsequently kept fixed during evaluation on the independent test subset. Alarm state was reset at the beginning of each recording session, and no temporal state was transferred between independent sessions. Stateful performance was evaluated using precision, recall, F1-score, FP/100, and false-positive burst rate.

### 2.7. Edge-Oriented Deployment Analysis

Although the CY8CKIT-062S2-AI platform integrates both radar and audio sensing hardware, it was not used as the sole end-to-end deployment platform in this study for two practical reasons. First, within the acquisition workflow used in this work, the radar stream had to be handled separately from the audio stream, which prevented the required simultaneous radar–audio acquisition configuration from being implemented on a single board. Consequently, two closely colocated CY8CKIT-062S2-AI boards were used during data acquisition. Second, the complete processing pipeline selected in this study, including the trained classifiers, modality-specific preprocessing, score-level fusion, and stateful alarm logic, was not directly deployable through the available embedded workflow without additional model conversion or custom implementation.

For these reasons, a Raspberry Pi 5 (Raspberry Pi Ltd., Cambridge, UK) was selected as the edge-computing platform for deployment evaluation. This choice allowed the complete processing chain to be executed without modifying the trained models, decision thresholds, or temporal alarm parameters, while preserving the numerical implementation used during model development. The validation-selected window-level and stateful configurations of the four detection branches were therefore exported for replay-based execution on the Raspberry Pi 5. The deployment package contained the trained classifiers, preprocessing specifications, decision thresholds, stateful alarm parameters, and the aligned independent test subset. The target platform used a 64-bit aarch64 Linux environment with four logical processor cores and Python 3.13.5. No retraining, threshold adjustment, or temporal-parameter adaptation was performed on the Raspberry Pi; the objective of the deployment experiment was to verify cross-platform reproducibility and quantify the computational requirements of the fixed processing chains.

#### 2.7.1. Replay-Based Deployment Protocol

The exported window-level configurations were the radar MLP, audio MLP, calibrated Linear SVC early-fusion classifier, and MAX late fusion applied to the scores of the radar and audio MLPs. Stateful selection changed the unimodal radar and audio classifiers to Random Forest models, while the calibrated Linear SVC remained selected for early fusion. Importantly, the stateful late-fusion configuration did not replace the two unimodal MLP score generators with the Random Forest models: it retained MAX fusion of the radar-window MLP and audio-window MLP scores and subsequently applied the validation-selected exponential moving average, hysteresis, and persistence logic. This distinction permits the computational effect of temporal alarm processing to be separated from the larger model-footprint changes introduced by stateful unimodal model selection.

All branches were replayed on the same 1244 temporally aligned test windows used in the main evaluation. Prediction agreement with the reference development environment was checked at both score and decision levels. Computational execution was characterized using mean per-window latency, 95th-percentile (P95) latency, processing throughput, serialized model size, process memory, and device temperature. The timing benchmark was repeated five times, with 90 s cooling intervals between runs. In addition, a separate late-fusion replay reconstructed the audio input directly from the raw audio recordings while retaining the radar macro-detection input in its already windowed form. Consequently, the latter experiment represents raw-audio-to-decision replay with pre-windowed radar input rather than a full raw-radar sensor-to-alarm benchmark.

#### 2.7.2. Live Multimodal Sensor-to-Decision Evaluation Protocol

To complement the replay-based deployment analysis, a live proof-of-concept implementation was evaluated on the Raspberry Pi 5 using the two CY8CKIT-062S2-AI boards as simultaneous radar and audio sensing nodes. Radar macro-detection data and audio samples were streamed to the Raspberry Pi, where the same modality-specific preprocessing used during model development was applied and temporally corresponding 1.6 s observation windows were generated at a 0.8 s update interval.

The live experiment focused on the two multimodal configurations considered most relevant to the final sensing system: Early Fusion and Late Fusion. The Early Fusion pipeline generated a direct window-level decision from the concatenated radar and audio descriptors, whereas the Late Fusion pipeline combined the radar and audio classifier scores using the validation-selected MAX rule and subsequently applied the previously selected EMA, hysteresis, and persistence parameters to generate a stable alarm state. All trained models, preprocessing parameters, decision thresholds, fusion rules, and temporal parameters were kept unchanged from the offline experiments; no retraining or live-specific tuning was performed.

Live execution was evaluated using repeated 60 s runs under quiet and acoustically disturbed conditions. Per-window inference time and total processing time, including live preprocessing and decision generation, were recorded. The first processing window included one-time stream and runtime initialization and was therefore treated as a warm-up sample rather than as representative steady-state latency. Because the live trials were not accompanied by an independently synchronized event-level ground truth, this experiment was used to assess continuous sensor-to-decision feasibility and timing behavior rather than to provide an additional classification-accuracy estimate.

### 2.8. External Cross-Environment Evaluation

To assess the robustness of the proposed system after relocation, an additional external evaluation was performed in Room 2, located in a different building. Room 2 had approximately the same dimensions as Room 1 (about 5 × 5 m), but differed in its internal access geometry, with the door located on the wall opposite the window. The wall construction, flooring, door, window, and furniture materials also differed from those of Room 1, resulting in modified radar-reflection and acoustic conditions.

The sensing assembly was installed at the same height as in Room 1 and oriented so that both the door and the window remained within the monitored region. The experiment therefore evaluated transfer of the frozen detection pipeline to a room of comparable size but with different internal geometry and material characteristics, while preserving the principal sensor-mounting conditions.

Ten additional recording sessions were acquired in Room 2. The scenarios included single- and multi-person intrusion events, slow and rapid entries, access through both the door and the window, door-handle and impact interactions, falling-object events, and external acoustic disturbances including human voices. After radar–audio alignment, the external test set contained 966 paired windows, comprising 423 positive and 543 negative windows. No radar–audio label disagreement was observed, and the median absolute temporal difference between paired samples was approximately 50 ms.

Room 2 was used exclusively for external evaluation. The classifiers, preprocessing parameters, feature scaling, decision thresholds, late-fusion rule, and stateful alarm parameters obtained from Room 1 were kept unchanged. No retraining, threshold adjustment, or environment-specific calibration was performed using Room 2 data. The recordings were processed on the Raspberry Pi 5 using the same frozen processing configuration employed for the Room 1 deployment evaluation.

## 3. Results

All model configurations, decision thresholds, and temporal alarm parameters were selected exclusively using the development data, while the independent test subset was reserved for final evaluation. Results are presented for model selection and window-level discrimination, stateful alarm behavior, late-fusion comparison, Raspberry Pi deployment, live sensor-to-decision execution, and cross-environment evaluation.

### 3.1. Candidate Model Selection

Candidate classifiers were evaluated separately for the radar-only, audio-only, and early-fusion branches using the validation-based model-selection protocol described in Section 2.5. The purpose of this stage was to identify the most suitable classifier for each feature representation before any final evaluation on the independent test subset. Model selection was based primarily on validation F1-score, while precision, recall, and false-positive behavior were also considered to avoid selecting configurations that achieved a favorable aggregate score at the expense of excessive missed detections or nuisance alarms. Because the radar, audio, and early-fusion representations differ substantially in dimensionality and statistical structure, the same classifier family was not assumed to be optimal for all branches.

Table 3 shows that the MLP provided the highest validation F1-score for both unimodal branches and was therefore retained for radar-only and audio-only evaluation. For early fusion, the calibrated Linear SVC achieved the highest validation F1-score and recall, whereas the tree-based alternatives generally reduced false positives at the cost of lower recall. These selections are consistent with the validation criterion defined in Section 2.5, which prioritized F1-score while also considering recall, precision, and false-positive behavior. Only the selected configurations were subsequently evaluated on the independent test subset.

### 3.2. Window-Level Evaluation

Following the validation-based model selection described in Section 3.1, the retained radar-only, audio-only, and early-fusion classifiers were evaluated on the independent test subset. The radar and audio MLP classifiers were also used as the fixed unimodal inputs to the late-fusion stage. Four score-level fusion strategies—AVG, MAX, PROD, and LR—were evaluated using the same aligned test windows. All configurations were therefore compared on the same set of 1244 windows, comprising 624 positive and 620 negative samples.

Table 4 reports the window-level performance of the three validation-selected learning branches together with the four late-fusion alternatives. Decision thresholds were determined from validation data and subsequently kept fixed for test evaluation.

The validation-selected decision thresholds were 0.693 for radar-only detection, 0.970 for audio-only detection, and 0.406 for early fusion. For late fusion, the corresponding thresholds were 0.988 for MAX, 0.205 for PROD, 0.530 for AVG, and 0.703 for LR.

Among the unimodal configurations, radar-only detection provided the stronger overall result, achieving an F1-score of 0.842 compared with 0.789 for audio-only detection. The difference was mainly associated with recall, which increased from 0.678 for audio to 0.764 for radar. Audio nevertheless produced fewer false-positive windows, indicating that its lower F1-score was primarily caused by missed intrusion-related events rather than excessive false detections.

Early fusion increased recall to 0.785 and F1-score to 0.854, exceeding both unimodal branches and confirming that the two sensing modalities provide complementary information. However, its false-positive rate increased to 5.48 per 100 negative windows, indicating that feature-level combination did not provide the best overall balance between sensitivity and nuisance detections. The higher false-positive rate should not be interpreted as a consequence of inaccurate spatial registration, since no point-wise spatial correspondence is imposed between the two modalities. Rather, the early-fusion classifier operates on temporally aligned but strongly compressed statistical descriptors. This representation intentionally sacrifices part of the fine within-window structure of the original sensor streams in exchange for lower dimensionality and reduced model complexity. Consequently, nuisance-related evidence present in either compact descriptor can influence the joint decision boundary. In the present test set, this produced a small gain in recall relative to radar-only detection (0.785 versus 0.764), but also a modest increase in false-positive windows (5.48 versus 5.16 per 100 negative windows). These results indicate that direct descriptor concatenation did not achieve the same sensitivity–specificity balance as score-level fusion.

All four late-fusion strategies improved some aspects of the multimodal decision compared with the unimodal branches, but their behavior differed substantially. AVG achieved the highest PR-AUC and ROC-AUC, indicating strong threshold-independent score discrimination, while PROD produced the lowest false-positive rate but at the expense of recall. LR provided an intermediate compromise but did not exceed the best deterministic fusion rules in terms of F1-score.

The MAX rule achieved the highest window-level F1-score of 0.897 and the highest recall of 0.848, together with a precision of 0.953 and 4.19 false-positive windows per 100 negative windows. It therefore provided the strongest operating-point balance among the evaluated late-fusion alternatives. Relative to radar-only detection, MAX increased F1-score by 5.5 percentage points and recall by 8.4 percentage points while reducing the false-positive rate from 5.16 to 4.19. These results support the use of score-level fusion when intrusion evidence may be expressed strongly in either radar or audio rather than simultaneously in both modalities.

To further characterize generalization under the limited-data setting, the selected window-level configurations were also evaluated on the training, validation, and independent test subsets using the validation-selected operating thresholds. The radar-only MLP achieved F1-scores of 0.938, 0.884, and 0.842 on the training, validation, and test subsets, respectively. The corresponding values were 0.858, 0.726, and 0.789 for the audio-only MLP; 0.944, 0.882, and 0.854 for early fusion; and 0.909, 0.824, and 0.897 for late-fusion MAX. The resulting train-to-test F1 differences were 0.096, 0.069, 0.090, and 0.012, respectively.

The aggregate comparison of precision, recall, F1-score, and false-positive behavior is shown in Figure 3, while the corresponding confusion matrices of the four selected configurations are presented in Figure 4.

### 3.3. Stateful Alarm Evaluation

Stateful model selection was performed independently from the window-level selection because temporal smoothing, hysteresis, and persistence can substantially modify the operating behavior of a classifier. Consequently, the model providing the highest isolated window-level F1-score was not necessarily the most suitable configuration after temporal alarm processing. For each candidate model, the EMA coefficient, activation and deactivation thresholds, and persistence parameters were optimized using the validation sequences. Selection considered the resulting stateful F1-score together with recall, false-positive windows, and false-positive alarm bursts, with particular emphasis on reducing repeated nuisance activations while preserving sufficient sensitivity to intrusion-related events. This procedure selected Random Forest for both radar-only and audio-only stateful operation, while the calibrated Linear SVC remained selected for early fusion and MAX remained the selected late-fusion rule. All selected configurations and temporal parameters were fixed before evaluation on the independent test subset. The resulting stateful test performance is summarized in Table 5.

The stateful validation results further support these selections. For radar-only detection, the temporally optimized Random Forest achieved a validation F1-score of 0.856, compared with 0.848 for the temporally optimized MLP, with both configurations satisfying the predefined radar feasibility constraints. For audio-only detection, the corresponding validation F1-scores were 0.804 for Random Forest and 0.789 for MLP. No audio candidate simultaneously satisfied both predefined feasibility constraints; therefore, the fallback ranking defined in Section 2.6 was applied, which retained the Random Forest configuration.

The change from MLP to Random Forest in the radar and audio branches indicates that the classifier providing the highest isolated window-level F1-score did not necessarily yield the most favorable sequential alarm behavior. In contrast, the early-fusion Linear SVC and the MAX late-fusion rule remained the preferred configurations after temporal processing. Among the four stateful branches, late fusion achieved the highest F1-score and recall while maintaining a low false-positive rate.

Figure 5 visualizes the stateful performance and false-alarm behavior across branches, while Figure 6 shows the resulting confusion matrices. The temporal parameters were selected independently for each branch; therefore, Figure 5 does not represent performance under a common hysteresis band. The 0.01 hysteresis band reported in Section 3.4 applies specifically to the selected late-fusion MAX configuration.

The radar-only stateful configuration, based on Random Forest, exhibited the most conservative behavior. It reached a precision of 0.969 and reduced the false-positive rate to 1.77 per 100 negative windows, but recall decreased to 0.559, producing an F1-score of 0.709. The low recall reflects the conservative behavior of the validation-selected Random Forest together with its temporal alarm parameters; because the window-level radar branch used a different classifier, the value of 0.559 should not be interpreted as a recall reduction caused by temporal smoothing alone.

The selected audio-only Random Forest configuration achieved an F1-score of 0.784, with a precision of 0.928 and a recall of 0.679. Its false-positive rate remained above five per 100 negative windows, indicating that temporal aggregation alone could not fully resolve the ambiguity between intrusion-related sounds and the acoustic hard-negative scenarios.

For early fusion, the stateful alarm reached a precision of 0.967, a recall of 0.715, and an F1-score of 0.822. The false-positive rate decreased from 5.48 at the window level to 2.42 per 100 negative windows, corresponding to a reduction of approximately 56%. This improvement in alarm stability was accompanied by a decrease in recall, showing the expected trade-off between nuisance-alarm suppression and event sensitivity.

The late-fusion MAX alarm achieved the strongest overall balance, with a precision of 0.970, a recall of 0.819, and an F1-score of 0.888. Its false-positive rate was 2.58 per 100 negative windows, while the false-positive burst rate was 1.29 per 100 negative windows. Late fusion therefore retained substantially more intrusion-related activity than the other stateful branches while maintaining limited false-positive window coverage and a reduced number of false-alarm bursts.

### 3.4. Effect of Temporal Processing on the Selected Late-Fusion System

Unlike the unimodal comparisons above, this analysis keeps the underlying radar and audio MLP classifiers and the MAX fusion rule unchanged. The window-level and stateful results can therefore be compared directly to isolate the effect of the temporal alarm processing.

For the final late-fusion configuration, the MAX score was processed using an exponential moving average coefficient of 0.6, a minimum activation persistence of three consecutive windows, a minimum deactivation persistence of one window, and a hysteresis band of 0.01. The activation and deactivation thresholds selected on the validation subset were 0.872 and 0.862, respectively. The effect of this temporal processing on the selected late-fusion configuration is summarized in Table 6. Here, TN, FP, FN, and TP denote true-negative, false-positive, false-negative, and true-positive window decisions, respectively.

The stateful logic reduced the number of false-positive windows from 26 to 16 and increased the number of true-negative decisions from 594 to 604, corresponding to a 38.5% reduction in false-positive windows. At the same time, true-positive decisions decreased from 529 to 511, while false negatives increased from 95 to 113.

This trade-off increased precision from 0.953 to 0.970, while recall decreased from 0.848 to 0.819. The F1-score decreased by less than one percentage point, from 0.897 to 0.888. Thus, the temporal alarm mechanism removed a substantial proportion of false activations without strongly degrading the overall detection balance.

Compared with the stateful early-fusion branch, the selected late-fusion alarm improved F1-score by approximately 6.6 percentage points and recall by 10.4 percentage points. Precision remained similar, increasing from 0.967 to 0.970, while the false-positive rate increased only slightly from 2.42 to 2.58 per 100 negative windows. In confusion-matrix terms, late fusion generated only one additional false-positive window but correctly detected 65 additional positive windows, increasing the number of true positives from 446 to 511.

### 3.5. Comparison of Late-Fusion Rules

The score distributions of the four late-fusion strategies were further compared using precision–recall and receiver-operating-characteristic curves, as shown in Figure 7.

The curves complement the operating-point results reported in Table 4 by illustrating the threshold-independent discrimination behavior of AVG, MAX, PROD, and LR.

During stateful validation, both MAX and AVG satisfied the predefined late-fusion feasibility constraints of recall ≥ 0.75 and FP/100 ≤ 5.0. Because the validation metrics favored different candidate configurations, feasible late-fusion configurations were ranked using a validation-only composite score combining F1-score, recall, precision, FP/100, and false-positive burst rate. MAX achieved the highest validation selection score and was therefore retained as the final configuration; its validation F1-score was also slightly higher than that of AVG (0.878 versus 0.873). On the independent test subset, AVG subsequently produced a numerically higher stateful F1-score of 0.914 compared with 0.888 for MAX. This post-selection result was not used to replace the validation-selected MAX rule, thereby preserving the separation between model selection and final evaluation and avoiding test-set adaptation.

### 3.6. Edge-Oriented Deployment Considerations

#### 3.6.1. Replay-Based Deployment Results

The exported processing chains reproduced the reference detection behavior on the Raspberry Pi 5 without platform-specific retuning. Decision-level agreement with the reference development environment was 1.0 for all eight evaluated outputs, comprising the four window-level and four stateful configurations.

Continuous-score differences remained small and did not change any binary decision. Accordingly, the Raspberry Pi replay preserved the test performance already reported for the corresponding branches, including an F1-score of 0.897 for window-level late fusion and 0.888 after stateful temporal processing. Table 7 summarizes the repeated computational benchmark. The late-fusion MAX branch required 0.1837 ± 0.0011 ms per stored feature window at the window level and 0.1836 ± 0.0008 ms after stateful processing. The nearly unchanged execution time indicates that the EMA, hysteresis, and persistence operations add negligible computational overhead relative to the two unimodal score generators.

The repeated timing results show that all tested configurations operate with a large computational margin relative to the 0.8 s stride between consecutive windows. Even the slowest mean branch execution remained below 0.3 ms per stored feature window. These values should be interpreted as replay-time processing costs after feature-window preparation, rather than as complete live-sensor acquisition latency.

A separate late-fusion experiment therefore reconstructed the acoustic frontend directly from the raw WAV recordings. The first pass required 2.0540 ms per generated audio window and 2.2341 ms from raw audio to the late window-level decision, while the corresponding stateful path required 2.2357 ms. After file, library, and cache warm-up, the second pass required 1.1137 ms for the raw-audio frontend and approximately 1.30 ms per window for both the window-level and stateful late-fusion decisions. The reconstructed audio features remained numerically consistent with the stored reference representation, and the reproduced detection metrics were unchanged. Radar input remained pre-windowed in this experiment; therefore, these measurements are not reported as full raw-radar sensor-to-alarm latency.

Model footprint further differentiates the window-level and stateful selections. As summarized in Table 8, the Random Forest models selected for the stateful radar-only and audio-only branches were substantially larger than the corresponding window-level MLP models. In contrast, the selected late-fusion system requires only the two window-level MLP classifiers, with a combined serialized payload of approximately 1.16 MB; MAX score fusion and the temporal alarm logic do not require an additional learned model.

The complete Python replay harness reached a substantially larger process-memory footprint because all exported models, test arrays, and runtime libraries were loaded by the benchmark process. This value was therefore not treated as the intrinsic memory requirement of the selected late-fusion deployment. Across the repeated measurements, device temperature remained within the range observed for the benchmark runs, and the separate late-fusion replay reached 52.9 °C. No prolonged thermal-stress or throttling experiment was performed.

Overall, the replay-based Raspberry Pi 5 measurements confirm cross-platform reproducibility, compact model storage, and substantial computational margin. Late score-level fusion combines the strongest detection performance with a compact classifier payload and negligible additional cost for MAX fusion and stateful temporal processing. Complementary live sensor-to-decision feasibility is evaluated in Section 3.6.2, while longer-duration thermal, memory, and stability characterization remains future work.

#### 3.6.2. Live Multimodal Sensor-to-Decision Proof-of-Concept Results

The live multimodal implementation was evaluated using four 60 s benchmark runs for each of the two selected multimodal pipelines: Early Fusion and Stateful Late MAX. Each run produced 75 decisions spanning approximately 59.2 s, consistent with the 0.8 s window stride.

The first processed window exhibited a substantially higher one-time latency of approximately 1.07–1.12 s due to stream and runtime initialization and was therefore excluded from the steady-state timing statistics. Across the remaining windows, Early Fusion required an average total processing time of 48.53 ms per window, with an average 95th-percentile latency of 64.01 ms. Stateful Late MAX required 46.50 ms per window on average, with an average 95th-percentile latency of 59.83 ms. The corresponding mean model-inference times were 1.79 ms for Early Fusion and 1.23 ms for Stateful Late MAX.

The maximum steady-state processing times observed across all runs were 125.70 ms for Early Fusion and 146.84 ms for Stateful Late MAX, both remaining well below the 0.8 s interval between consecutive decisions. The larger total-processing value reflects the inclusion of sensor-window retrieval, feature extraction, fusion, and stateful processing, whereas the 1.23 ms value refers only to classifier inference. These measurements show that both multimodal configurations sustained continuous live processing on the Raspberry Pi 5 without computational backlog. The steady-state timing results for both multimodal pipelines are summarized in Table 9.

**Table 9:** Steady-state timing results for the live multimodal Raspberry Pi 5 implementation.

Configuration	Mean Total Processing [ms/Window]	Mean P95 [ms]	Mean Inference [ms/Window]	Maximum Steady-State
Early fusion	48.53	64.01	1.79	125.70
Stateful Late fusion (MAX)	46.50	59.83	1.23	146.84

Values represent the average results obtained across four 60 s runs for each pipeline. The first processed window of each run was excluded from the steady-state statistics because it included one-time stream and runtime initialization overhead.

### 3.7. Cross-Environment Evaluation

The frozen configurations selected using Room 1 were additionally evaluated on the 966 aligned radar–audio windows acquired in Room 2, comprising 423 positive and 543 negative windows. To maintain direct comparability with the original test evaluation, the same performance indicators were used. No retraining, feature-scaling adaptation, decision-threshold adjustment, fusion-rule modification, or stateful-parameter tuning was performed using Room 2 data. Table 10 reports the window-level results obtained in the second environment.

At window level, late-fusion MAX achieved the highest F1-score (0.864), with a precision of 0.818, a recall of 0.915, and 15.84 false-positive windows per 100 negative windows. Radar-only detection produced a similar F1-score of 0.862 and the highest recall (0.972), together with the highest PR-AUC and ROC-AUC, but a higher false-positive rate of 22.10. Audio-only and early fusion reached F1-scores of 0.741 and 0.800, respectively. Compared with Room 1, late-fusion MAX changed from 0.897 to 0.864 in F1-score, while recall increased from 0.848 to 0.915 and precision decreased from 0.953 to 0.818.

To examine how the fixed decision thresholds selected using Room 1 validation data influenced cross-environment performance, Figure 8 compares the positive- and negative-class window-level score distributions in Rooms 1 and 2. The most pronounced change is observed in the negative-class distributions, which shift toward higher intrusion scores in Room 2. For late-fusion MAX, the median negative-class score increased from 0.108 in Room 1 to 0.598 in Room 2, while the proportion of negative windows exceeding the fixed threshold increased from 4.19% to 15.84%. Early fusion showed an even larger increase in threshold crossings, from 5.48% to 27.99%. The corresponding increases were from 5.16% to 22.10% for radar-only detection and from 4.03% to 18.23% for audio-only detection. These results indicate that the higher Room 2 false-positive rates are primarily associated with an upward shift in the negative-class score distributions relative to the fixed Room 1 operating thresholds.

Table 11 reports the corresponding results after applying the stateful alarm processing with the temporal parameters selected exclusively from Room 1 validation data.

With the frozen stateful parameters, late fusion retained the highest F1-score (0.859) and recall (0.939). Early fusion reached 0.820, followed by audio-only at 0.756 and radar-only at 0.708. For late fusion, stateful processing increased recall from 0.915 to 0.939 but also increased FP/100 from 15.84 to 19.15, while F1 changed only from 0.864 to 0.859.

## 4. Discussion

The results presented in Section 3 indicate that the main benefit of the proposed system arises from the complementary behavior of radar and audio rather than from either modality alone. Radar provided the stronger unimodal window-level result, with an F1-score of 0.842, while audio reached 0.789 and was mainly limited by lower recall. Early fusion improved the F1-score to 0.854, confirming that the two sensing channels contain complementary information. The strongest operating-point result was obtained with score-level late fusion, where the MAX rule reached an F1-score of 0.897. This behavior is consistent with the alarm-oriented formulation of the task, since an intrusion-related event may generate strong evidence in either radar or audio, and MAX preserves the strongest modality response. Radar primarily contributes motion-related evidence, whereas audio adds complementary cues from footsteps, impacts, and access interactions; this complementarity is reflected by the increase from F1 = 0.842 for radar-only and 0.789 for audio-only to 0.897 for late-fusion MAX. This interpretation is also aligned with previous work emphasizing the value of multimodal sensing and sensor-data fusion for indoor human monitoring and security-oriented event detection [1,10].

The comparison across training, validation, and independent test subsets provides additional evidence regarding generalization under the limited-data setting. As expected, radar-only and early fusion exhibited some reduction in F1-score from training to test, with differences of 0.096 and 0.090, respectively. However, neither configuration showed a collapse in performance on the independent test data. The audio-only branch achieved a higher test F1-score than validation F1-score, while late-fusion MAX showed the smallest train-to-test difference, only 0.012, and also performed better on the test subset than on validation. This behavior, together with the session-disjoint partitioning and group-aware model-selection procedure, indicates that the reported performance is not dominated by substantial overfitting to the training recordings.

The distinction between window-level and stateful evaluation proved important. For the selected late-fusion configuration, temporal smoothing, hysteresis, and persistence reduced the number of false-positive windows from 26 to 16, corresponding to a 38.5% reduction, while precision increased from 0.953 to 0.970. This improvement was accompanied by a moderate decrease in recall from 0.848 to 0.819 and a small F1-score reduction from 0.897 to 0.888. The stateful stage should therefore be interpreted as an operational trade-off that suppresses nuisance activations rather than as a post-processing step that necessarily improves all classification metrics. More broadly, the emphasis on sequential decision behavior is consistent with prior edge-oriented studies addressing real-time acoustic event detection and temporal aggregation of acoustic events [12].

Stateful model selection also showed that the classifier providing the best independent-window result is not always the most suitable for sequential alarm operation. Random Forest models were selected for the radar-only and audio-only stateful branches, while the calibrated Linear SVC remained selected for early fusion and MAX remained selected for late fusion. This supports the use of temporal alarm behavior as part of model selection when the final application is sequential. Importantly, although AVG later produced a numerically higher stateful F1-score on the independent test subset, MAX was retained because it had been selected on validation data, thereby avoiding test-set adaptation.

The Raspberry Pi 5 deployment experiments support practical feasibility at two complementary levels. First, replay-based execution reproduced the reference decisions with complete decision-level agreement and showed that the selected late-fusion classifier payload remained compact. Second, the live proof-of-concept demonstrated continuous acquisition and processing of the radar and audio streams from the two sensing boards on the Raspberry Pi 5. Early Fusion and Stateful Late MAX required mean total processing times of 48.53 ms and 46.50 ms per window, respectively, while the maximum observed steady-state processing times remained below 150 ms. These values provide a substantial computational margin relative to the 0.8 s update interval and demonstrate that the proposed multimodal processing chain can operate continuously without computational backlog.

These results support a practical room-level architecture in which radar and audio sensing nodes provide complementary observations to a central edge-processing unit. The central unit performs preprocessing, classification, fusion, and temporal alarm processing and produces a binary room-level alarm state. The resulting binary state can trigger a local alarm actuator, such as a siren, or be forwarded to a higher-level security controller. The present experiments validate the sensing-to-decision processing chain; downstream actuator electronics and alarm-system integration were not evaluated.

The external Room 2 evaluation provides an additional perspective on deployment robustness. The second room was located in a different building and had dimensions comparable to Room 1, while differing in internal access geometry as well as wall, flooring, door, window, and furniture materials. Sensor height was kept the same, while the overall monitoring orientation was kept comparable to that used in Room 1. Despite these environmental changes and the absence of retraining or parameter adaptation, late-fusion MAX retained a window-level F1-score of 0.864 compared with 0.897 in Room 1. Recall increased from 0.848 to 0.915, whereas precision decreased from 0.953 to 0.818 and the false-positive rate increased from 4.19 to 15.84 per 100 negative windows. This suggests that intrusion sensitivity transferred more consistently than nuisance-alarm calibration across the two environments.

The stateful results reinforce this distinction. In Room 1, temporal processing reduced the late-fusion false-positive rate from 4.19 to 2.58 per 100 negative windows, whereas in Room 2 the same frozen temporal parameters increased the rate from 15.84 to 19.15 while recall increased from 0.915 to 0.939. This indicates that the learned detection and fusion functions and the temporal alarm calibration should be considered separately when transferring the system between indoor environments. MAX preserves strong intrusion evidence from either modality, while temporal smoothing, hysteresis, and persistence suppress isolated score excursions; however, this benefit diminishes when environmental changes produce sustained nuisance scores or shift the score distribution relative to the calibration environment.

The radar and acoustic devices are commercially available as standalone components and could be integrated on a dedicated sensing PCB, reducing the hardware redundancy and cost associated with the development-board prototype.

The CY8CKIT-062S2-AI nevertheless remains an attractive option for further system integration because the required radar and acoustic sensing hardware, embedded processing resources, wireless connectivity, and battery support are already available on the same platform. In the workflow used in this study, however, simultaneous acquisition of the required radar and audio streams and direct deployment of the complete selected processing pipeline on a single CY8CKIT-062S2-AI board were not available without additional custom implementation. If future platform and software support removes these limitations and expands the range of directly deployable models, the complete sensing and decision pipeline could potentially be consolidated onto a single embedded platform, eliminating both the additional acquisition board and the Raspberry Pi-class processing unit.

The study remains subject to several limitations. Model development and parameter selection were based on data acquired in one approximately 5 × 5 m room and on a limited number of independent recording sessions. The additional Room 2 experiment extends the evaluation to a second room located in a different building and characterized by different wall, flooring, door, window, and furniture materials; however, it represents only a preliminary cross-environment assessment based on ten additional sessions. Sensor height was intentionally kept the same between the two environments, while the overall monitoring orientation remained comparable between the two environments, and the increased false-positive rate observed in Room 2 indicates that nuisance-alarm behavior remains sensitive to environmental changes. Broader validation should therefore include additional buildings, room geometries, sensor positions and orientations, and longer-duration recordings under a wider range of environmental disturbances. Although non-front-facing intrusion cases were included in the dataset, the radar standard-deviation profiles were not evaluated separately as a function of subject orientation or approach angle; such an analysis remains for future work.

A further limitation concerns the scope of the live deployment evaluation. Although continuous radar–audio sensor-to-decision operation was demonstrated on the Raspberry Pi 5, the live experiments consisted of repeated short-duration benchmark runs and were not accompanied by an independent, synchronized event-level ground truth. They therefore establish live processing and timing feasibility rather than providing an additional detection-accuracy evaluation. Future work should include longer-duration live operation, synchronized event-level validation, robustness to sensor-stream interruption, direct alarm-actuator integration, and more detailed power and memory characterization.

## 5. Conclusions

This study investigated a limited-data radar–audio framework for camera-free indoor intrusion-related alarm detection, with particular emphasis on multimodal complementarity, operational alarm behavior, and edge-oriented deployment. Radar-only, audio-only, feature-level early-fusion, and score-level late-fusion strategies were evaluated using session-disjoint development and test data, thereby preventing correlated windows from the same recording from being shared across subsets.

The results obtained in the original acquisition environment confirmed the complementary behavior of the two sensing modalities. Radar provided the stronger unimodal performance, while score-level late fusion using the validation-selected MAX rule achieved the strongest overall window-level operating point, with an F1-score of 0.897. Temporal alarm processing based on exponential smoothing, hysteresis, and persistence reduced nuisance activations in this environment, lowering the false-positive rate from 4.19 to 2.58 per 100 negative windows while maintaining an F1-score of 0.888. These results show that window-level discrimination and operational alarm behavior provide complementary views of system performance and should be evaluated separately.

The deployment experiments further demonstrated both cross-platform reproducibility and live processing feasibility on a Raspberry Pi 5. Replay-based execution preserved the reference decisions without retraining or parameter adaptation, while the live implementation connected the two sensing boards directly to the Raspberry Pi 5 and sustained continuous multimodal processing. Mean total processing times were 48.53 ms per window for Early Fusion and 46.50 ms per window for Stateful Late MAX, with all steady-state measurements remaining well below the 0.8 s decision-update interval. These results support the use of a Raspberry Pi-class device as a central edge-processing unit receiving radar and audio observations and generating a room-level intrusion/no-intrusion state suitable for integration with a local or centralized alarm system.

An additional external evaluation was performed after transferring the frozen configuration to a second room of comparable dimensions located in a different building, with different internal access geometry, construction, and furnishing materials. Sensor height was kept the same, while the overall monitoring orientation was kept comparable to that used in the original room and the complete processing configuration remained frozen. Without retraining, threshold adjustment, or environment-specific calibration, the late-fusion MAX branch retained a window-level F1-score of 0.864 and a stateful F1-score of 0.859. The corresponding recall remained high, whereas precision and false-positive behavior degraded relative to the original environment. This indicates that the learned radar–audio detection and fusion functions transferred more consistently across environments than the temporal alarm calibration.

Overall, the results support score-level radar–audio fusion as a practical approach for edge-oriented intrusion detection under limited-data conditions. The combination of compact sensing, lightweight classification, multimodal score fusion, and stateful alarm processing provides a viable basis for a self-contained room-level monitoring system. At the same time, the increased nuisance-alarm rate observed in the second environment shows that broader deployment requires further validation across additional buildings, room geometries, sensor positions, environmental disturbances, and longer operating periods. Future work should extend the short-duration live proof-of-concept toward long-duration sensor-to-alarm operation with synchronized ground truth, direct actuator integration, additional room configurations, and robustness analysis under sensor-stream interruptions and broader environmental disturbances.

## Figures and Tables

**Figure 1:**
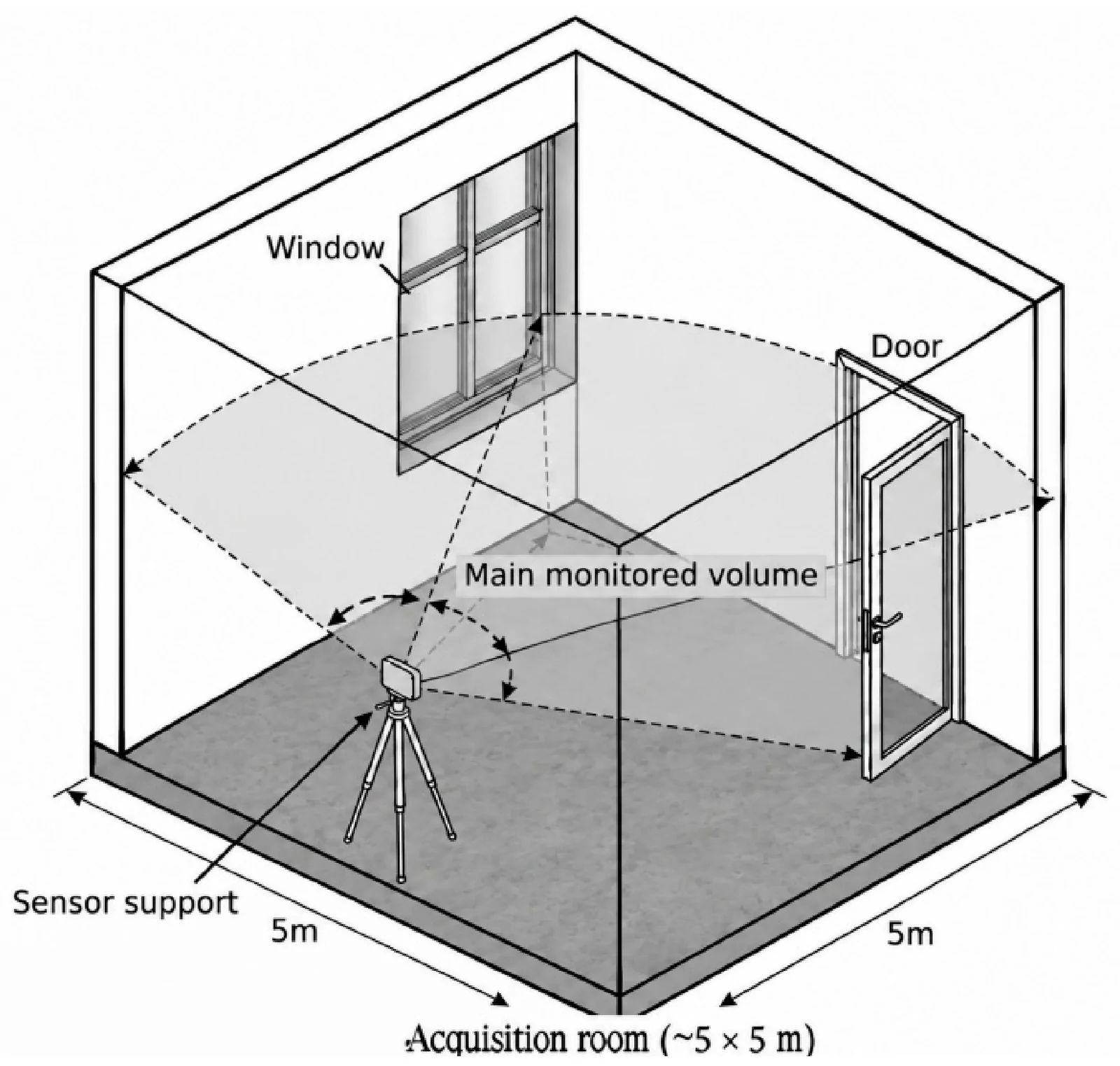
Schematic layout of Room 1 (approximately 5 × 5 m), showing the relative positions of the door, the window, the sensing location, and the main monitored region. The dashed arrows schematically indicate the sensing directions and the approximate extent of the monitored region, rather than measured detection boundaries.

**Figure 3:**
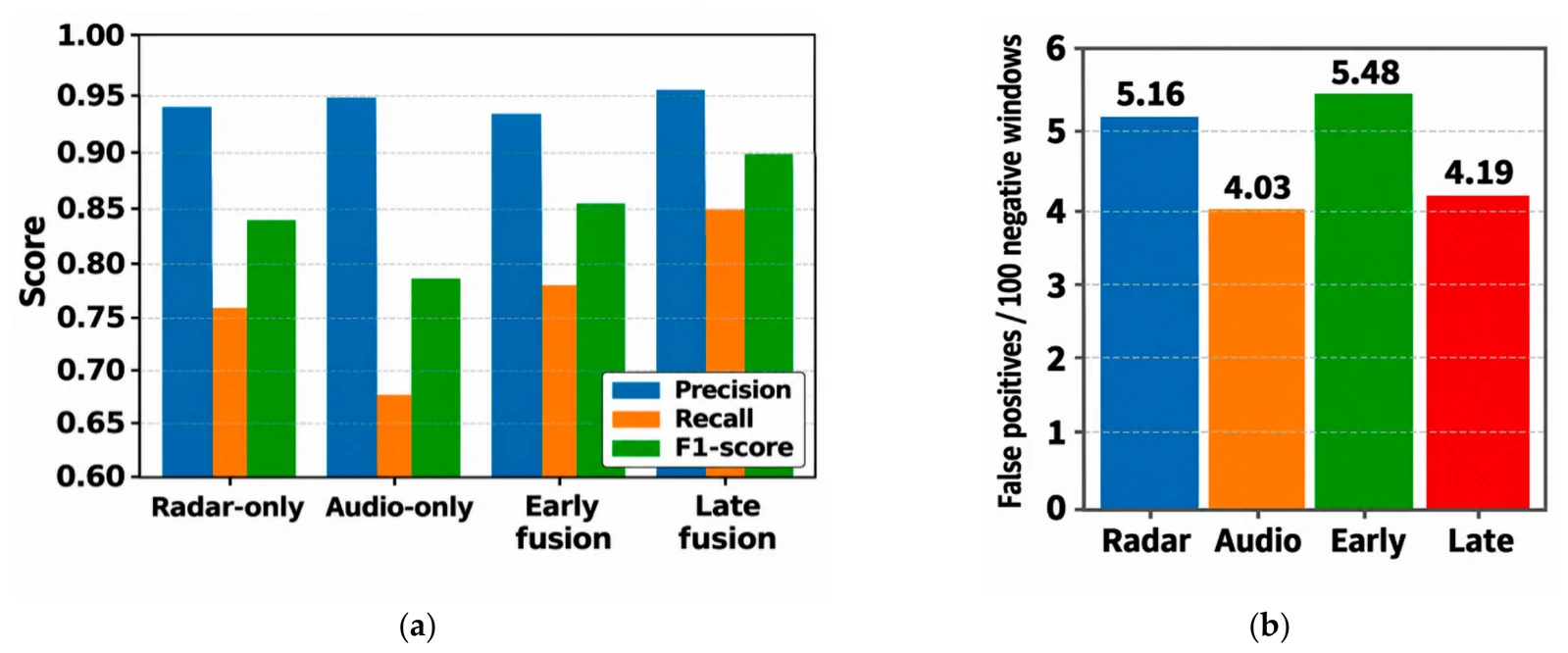
Window-level comparison on the independent test subset: (**a**) precision, recall, and F1-score for the selected radar-only, audio-only, early-fusion, and late-fusion configurations; (**b**) false-positive windows per 100 negative windows.

**Figure 4:**
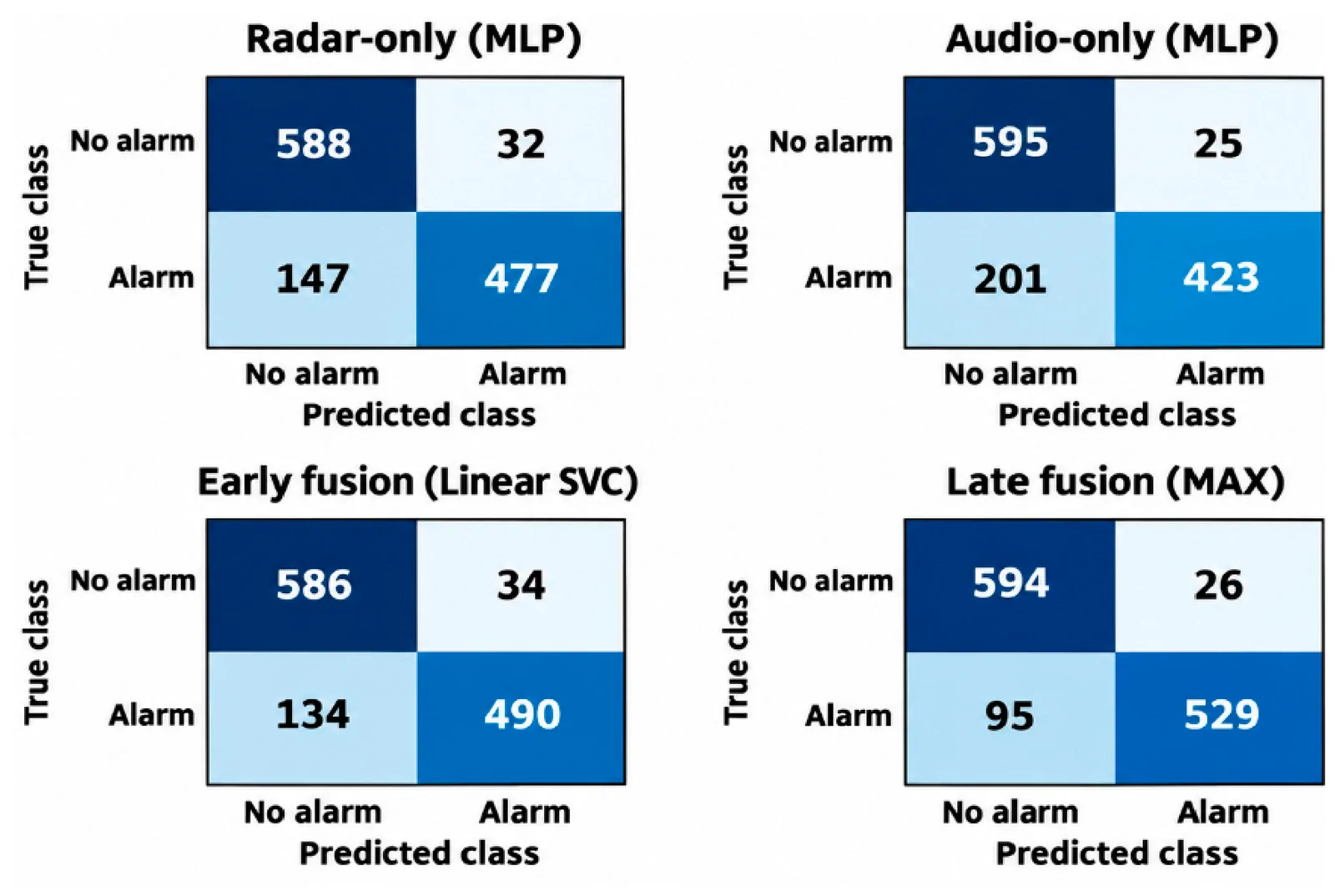
Confusion matrices of the selected window-level configurations on the test subset. Rows denote the true class and columns denote the predicted class; the positive class corresponds to an intrusion-related alarm event.

**Figure 5:**
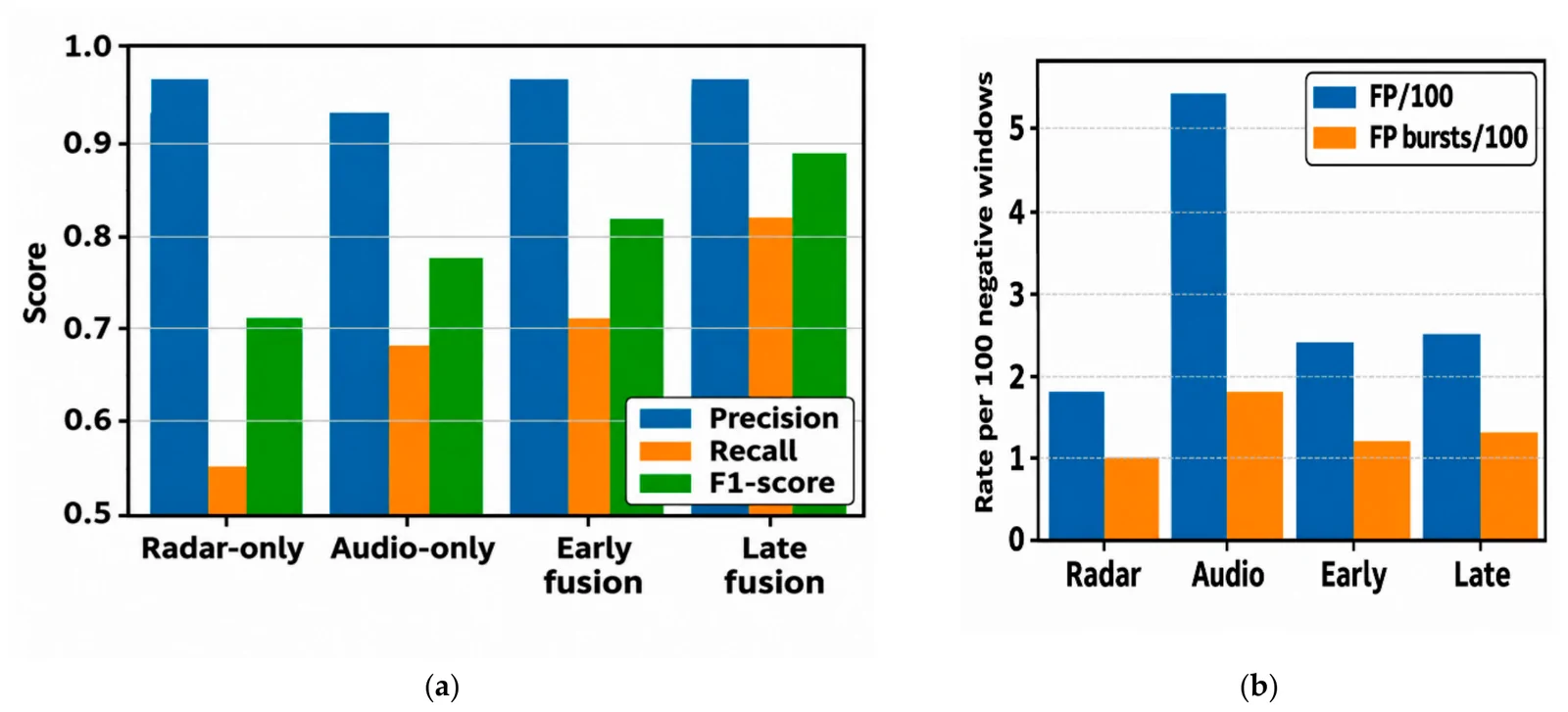
Stateful alarm comparison on the test subset: (**a**) precision, recall, and F1-score after temporal smoothing, hysteresis, and persistence; (**b**) false-positive windows and false-positive bursts per 100 negative windows.

**Figure 6:**
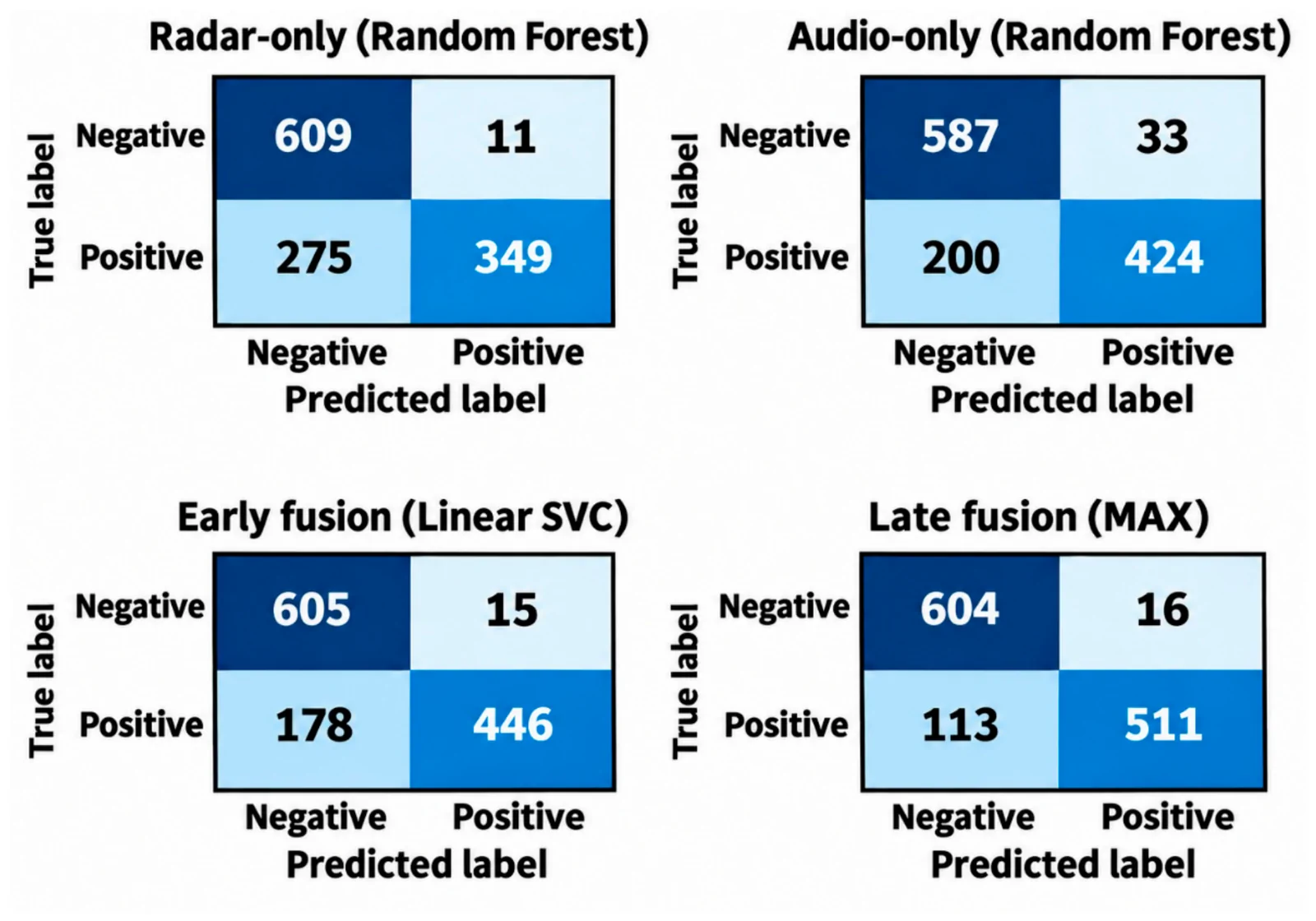
Confusion matrices of the validation-selected stateful alarm configurations on the test subset. The selected stateful model may differ from the corresponding window-level model because the two operating stages were tuned independently on validation data.

**Figure 7:**
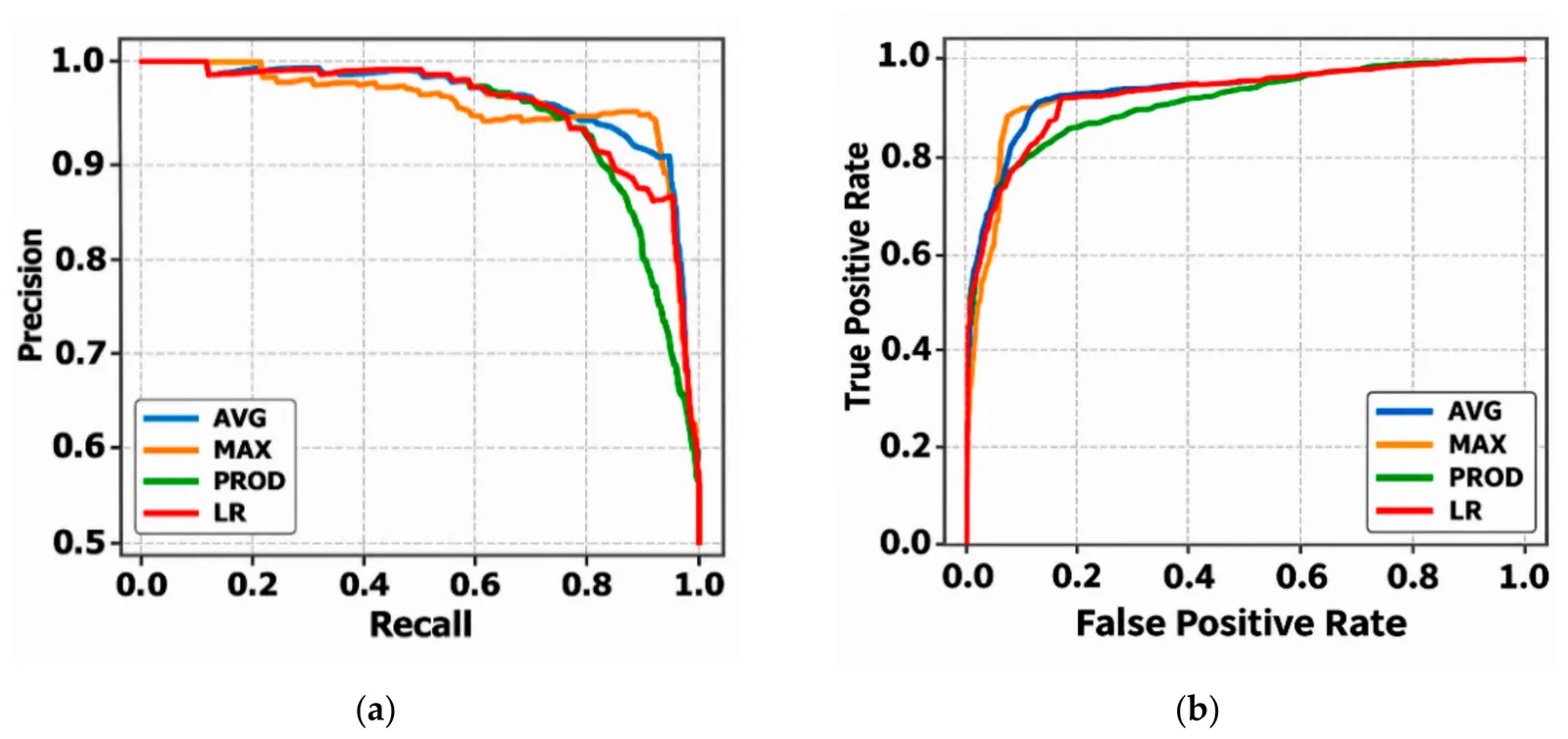
Window-level late-fusion performance on the test subset: (**a**) precision-recall curves and (**b**) receiver-operating-characteristic curves for average-score fusion (AVG), maximum-score fusion (MAX), probability-product fusion (PROD), and logistic-regression fusion (LR).

**Figure 8:**
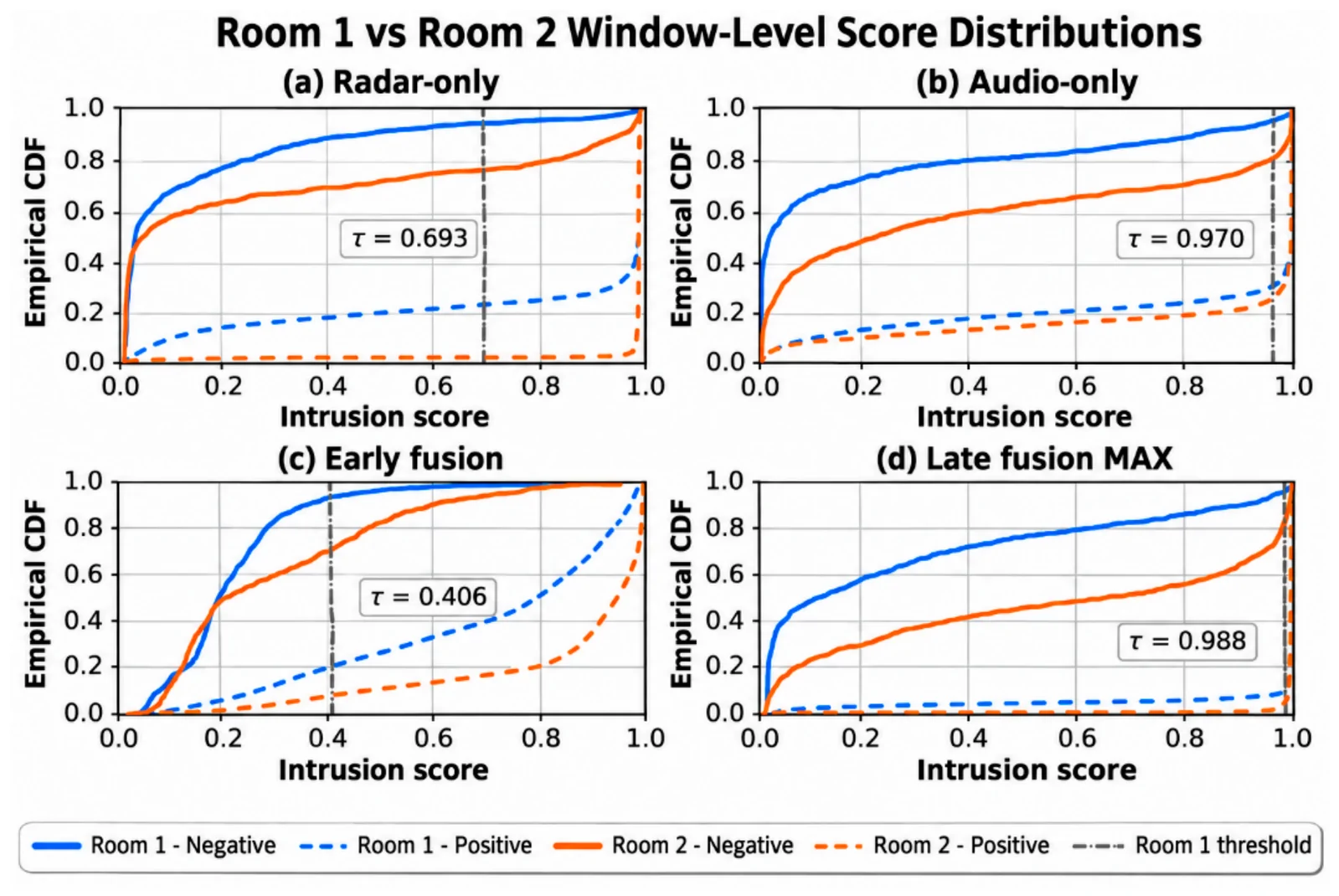
Empirical cumulative distributions of window-level intrusion scores for the positive and negative classes in Room 1 and Room 2: (**a**) radar-only; (**b**) audio-only; (**c**) early fusion; and (**d**) late-fusion MAX. Vertical dash-dot lines indicate the validation-selected Room 1 decision thresholds, which were kept fixed during Room 2 evaluation.

**Table 1:** Session-level partition and class distribution of the temporally aligned Room 1 radar–audio dataset used for model development and primary evaluation.

Total Windows	Negative Windows	Positive Windows	Sessions	Split
3518	1749	1769	42	Training
1317	655	662	14	Validation
1244	620	624	14	Test
6079	3024	3055	70	Total

**Table 2:** Stateful alarm parameter search spaces and feasibility constraints used during validation.

Branch	EMA Coefficient, α	Activation Persistence	Deactivation Persistence	Hysteresis Band	Feasibility Constraints
Radar-only	0.35, 0.50, 0.70, 0.85, 1.00	1	1	0, 0.01, 0.02	Recall ≥ 0.75; FP/100 ≤ 5.0
Audio-only	0.20, 0.40, 0.60, 0.90	2, 3, 4	1, 2	0.01, 0.02, 0.03, 0.07	Recall ≥ 0.75; FP/100 ≤ 5.0
Early fusion	0.70, 0.80, 0.90, 1.00	2, 3, 4	1, 4	0.025, 0.05	Recall ≥ 0.80; FP/100 ≤ 4.5
Late fusion—FP-prioritized	0.20, 0.30, 0.40, 0.50, 0.60, 0.80	1, 2, 3	1, 2	0.01, 0.02, 0.05	Recall ≥ 0.75; FP/100 ≤ 5.0
Late fusion—F1-prioritized	0.20, 0.30, 0.40, 0.50	1, 2, 3	1, 2	0.01, 0.02, 0.05	Recall ≥ 0.75; FP/100 ≤ 5.0

**Table 3:** Validation performance of the candidate classifiers evaluated for the radar-only, audio-only, and early-fusion branches.

Branch	Candidate Model	Precision	Recall	F1	FP/100	Selected
Radar-only	MLP	0.950	0.826	0.884	4.42	✓
	Logistic Regression	0.941	0.775	0.850	4.88	
	HistGradientBoosting	0.943	0.776	0.852	4.73	
	Random Forest	0.951	0.770	0.851	3.96	
	Extra Trees	0.951	0.754	0.848	3.96	
	Calibrated Linear SVC	0.937	0.449	0.607	3.05	
Audio-only	MLP	0.927	0.597	0.726	4.73	✓
	Logistic Regression	0.840	0.246	0.381	4.73	
	HistGradientBoosting	0.893	0.402	0.556	4.89	
	Random Forest	0.898	0.411	0.564	4.73	
	Extra Trees	0.903	0.450	0.601	4.89	
	Calibrated Linear SVC	0.780	0.166	0.274	4.73	
Early fusion	Calibrated Linear SVC	0.921	0.846	0.882	7.33	✓
	Logistic Regression	0.918	0.816	0.864	7.33	
	HistGradientBoosting	0.955	0.801	0.871	3.82	
	Random Forest	0.953	0.799	0.869	3.97	
	Extra Trees	0.968	0.778	0.863	2.60	
	MLP	0.924	0.811	0.864	6.72	

**Table 4:** Window-level test performance of the selected unimodal and early-fusion configurations and the evaluated late-fusion strategies.

FP/100	ROC-AUC	PR-AUC	F1	Recall	Precision	Configuration
5.16	0.919	0.932	0.842	0.764	0.937	Radar-only
4.03	0.901	0.915	0.789	0.678	0.944	Audio-only
5.48	0.919	0.938	0.854	0.785	0.935	Early fusion
4.19	0.956	0.956	**0.897**	**0.848**	**0.953**	**Late fusion** (MAX)
**3.71**	0.939	0.946	0.832	0.739	0.952	Late fusion (PROD)
5.16	**0.960**	**0.962**	0.883	0.832	0.942	Late fusion (AVG)
5.16	0.954	0.956	0.855	0.785	0.939	Late fusion (LR)

Note: Bold values indicate the best performance for each metric. Lower values are better for FP/100, whereas higher values are better for all other metrics.

**Table 5:** Performance of the stateful alarm outputs on the test subset.

FP Bursts/100	FP/100	F1	Recall	Precision	Configuration	Selected Model
**0.97**	**1.77**	0.709	0.559	0.969	Radar-only	Random Forest
1.77	5.32	0.784	0.679	0.928	Audio-only	Random Forest
1.13	2.42	0.822	0.715	0.967	Early fusion	Calibrated Linear SVC
1.29	2.58	**0.888**	**0.819**	**0.970**	**Late fusion**	**(MAX)**

Note: Bold values indicate the best performance for each metric. Lower values are better for FP/100 and FP bursts/100, whereas higher values are better for all other metrics.

**Table 6:** Effect of stateful alarm processing on the selected late-fusion MAX configuration.

TN/FP/FN/TP	FP/100	F1	Recall	Precision	Evaluation Stage
594/26/95/529	4.19	**0.897**	**0.848**	0.953	Window-level
604/16/113/511	**2.58**	0.888	0.819	**0.970**	Stateful alarm

Note: Bold values indicate the best performance for each metric. Lower values are better for FP/100, whereas higher values are better for all other metrics.

**Table 7:** Raspberry Pi 5 timing results over five repeated benchmark runs (mean ± standard deviation).

Branch	Evaluation	Mean Latency (ms/Window)	P95 Latency (ms)	Throughput (Windows/s)
Radar	Window-level	0.1655 ± 0.0058	0.1853 ± 0.0311	6047.3 ± 210.0
Audio	Window-level	0.0300 ± 0.0079	0.0359 ± 0.0187	34,829.6 ± 6874.1
Early fusion	Window-level	0.2107 ± 0.0064	0.2214 ± 0.0216	4750.1 ± 141.3
Late fusion (MAX)	Window-level	0.1837 ± 0.0011	0.1867 ± 0.0015	5442.7 ± 31.7
Radar	Stateful	0.2684 ± 0.0014	0.2745 ± 0.0018	3725.7 ± 18.8
Audio	Stateful	0.1089 ± 0.0019	0.1118 ± 0.0023	9187.6 ± 164.5
Early fusion	Stateful	0.2087 ± 0.0015	0.2121 ± 0.0021	4792.2 ± 34.6
Late fusion (MAX)	Stateful	0.1836 ± 0.0008	0.1862 ± 0.0011	5447.2 ± 24.8

**Table 8:** Serialized model sizes used in the Raspberry Pi 5 deployment package.

Component	Serialized Size (MB)
Radar window-level MLP	0.867
Audio window-level MLP	0.297
Early fusion calibrated Linear SVC	0.017
Radar stateful Random Forest	5.948
Audio stateful Random Forest	8.914
Late fusion classifier payload (two MLPs)	1.164

**Table 10:** Window-level cross-environment performance on Room 2 using the frozen Room 1 configurations and decision thresholds.

FP/100	ROC-AUC	PR-AUC	F1	Recall	Precision	Configuration
22.10	**0.975**	**0.976**	0.862	**0.972**	0.774	Radar-only
18.23	0.832	0.798	0.741	0.726	0.756	Audio-only
27.99	0.939	0.946	0.800	0.905	0.716	Early fusion
**15.84**	0.915	0.853	**0.864**	0.915	**0.818**	Late fusion (MAX)

Note: Bold values indicate the best performance for each metric. Lower values are better for FP/100, whereas higher values are better for all other metrics.

**Table 11:** Stateful cross-environment performance on Room 2 using the frozen Room 1 alarm parameters.

FP/100	FP Bursts/100	F1	Recall	Precision	Configuration
36.46	5.71	0.708	0.804	0.632	Radar-only
20.26	4.24	0.756	0.766	0.747	Audio-only
19.34	**3.68**	0.820	0.868	0.778	Early fusion
**19.15**	4.79	**0.859**	**0.939**	**0.792**	**Late fusion (MAX)**

Note: Bold values indicate the best performance for each metric. Lower values are better for FP/100 and FP bursts/100, whereas higher values are better for all other metrics.

## Data Availability

The complete radar–audio dataset used in this study, including the Room 2 recordings employed for cross-environment evaluation, is openly available through the GitHub repository Radar-Audio Intrusion Dataset for Privacy-Preserving Indoor Alarm Detection, release V1.0: https://github.com/vladueduared/Radar-Audio-Intrusion-Dataset/releases/tag/V1.0 (accessed on 1 October 2026). The release contains radar and audio recordings, event annotations, session-level metadata, and supporting documentation. No video recordings are included.

## Abbreviations

The following abbreviations are used in this manuscript:

AI	Artificial intelligence
AVG	Average-score fusion rule
EMA	Exponential moving average
FFT	Fast Fourier transform
FMCW	Frequency-modulated continuous wave
FN	False negative
FP	False positive
GT	Ground truth
IoT	Internet of Things
LR	Logistic regression
MAX	Maximum-score fusion rule
MLP	Multilayer perceptron
mmWave	Millimeter-wave
NFFT	Number of FFT points
P95	95th percentile
PR-AUC	Precision–recall area under the curve
PROD	Probability-product fusion rule
RF	Random Forest
ROC-AUC	Receiver operating characteristic area under the curve
STFT	Short-time Fourier transform
SVC	Support vector classifier
TN	True negative
TP	True positive
WAV	Waveform Audio File Format
